# Causal Inference in Time Series in Terms of Rényi Transfer Entropy

**DOI:** 10.3390/e24070855

**Published:** 2022-06-22

**Authors:** Petr Jizba, Hynek Lavička, Zlata Tabachová

**Affiliations:** 1 Faculty of Nuclear Sciences and Physical Engineering, Czech Technical University in Prague, Břehová 7, 115 19 Prague, Czech Republic; hynek.lavicka@fjfi.cvut.cz; 2 Complexity Science Hub Vienna, Josefstädter Straße 39, 1080 Vienna, Austria; zlata.tabachova@fjfi.cvut.cz

**Keywords:** Rényi entropy, Rényi transfer entropy, Rössler system, multivariate time series

## Abstract

Uncovering causal interdependencies from observational data is one of the great challenges of a nonlinear time series analysis. In this paper, we discuss this topic with the help of an information-theoretic concept known as Rényi’s information measure. In particular, we tackle the directional information flow between bivariate time series in terms of Rényi’s transfer entropy. We show that by choosing Rényi’s parameter α, we can appropriately control information that is transferred only between selected parts of the underlying distributions. This, in turn, is a particularly potent tool for quantifying causal interdependencies in time series, where the knowledge of “black swan” events, such as spikes or sudden jumps, are of key importance. In this connection, we first prove that for Gaussian variables, Granger causality and Rényi transfer entropy are entirely equivalent. Moreover, we also partially extend these results to heavy-tailed α-Gaussian variables. These results allow establishing a connection between autoregressive and Rényi entropy-based information-theoretic approaches to data-driven causal inference. To aid our intuition, we employed the Leonenko et al. entropy estimator and analyzed Rényi’s information flow between bivariate time series generated from two unidirectionally coupled Rössler systems. Notably, we find that Rényi’s transfer entropy not only allows us to detect a threshold of synchronization but it also provides non-trivial insight into the structure of a transient regime that exists between the region of chaotic correlations and synchronization threshold. In addition, from Rényi’s transfer entropy, we could reliably infer the direction of coupling and, hence, causality, only for coupling strengths smaller than the onset value of the transient regime, i.e., when two Rössler systems are coupled but have not yet entered synchronization.

## 1. Introduction

The time evolution of a complex system is usually recorded in the form of a time series. Time series analysis is a traditional field of mathematical statistics; however, the development of nonlinear dynamical systems and the theory of deterministic chaos have opened up new vistas in the analysis of nonlinear time series [1,2]. The discovery of the synchronization of chaotic systems [3] has changed the study of interactions and cooperative behavior of complex systems and brought new approaches to studying the relations between nonlinear time series [4]. During the process of synchronization, two systems can either mutually interact or only one can influence the other. In order to distinguish these two ways, and to find which system is the driver (“master”) and which is the response (“slave”) system, a number of approaches from the dynamical system theory have been proposed [5,6,7,8]. The aforementioned problem of synchronization can be seen as part of a broader framework known as *causality* or *causal relations between systems*, processes, or phenomena. The mathematical formulation of causality, in terms of predictability, was first proposed by Wiener [9] and formulated for the time series by Granger [10]. In particular, Granger introduced what is now known as *Granger causality*, which is a statistical concept of causality that is based on the evaluation of predictability in bivariate autoregressive models.

Extracting causal interdependencies from observational data is one of the key tasks in a nonlinear time series analysis. Apart from the linear Granger causality and various nonlinear extensions thereof [11,12,13], existing methods for this purpose include state-space-based approaches, such as conditional probabilities of recurrence [14,15,16], or information-theoretic quantities, such as conditional mutual information [17,18] and *transfer entropies* [2,19,20,21]. In particular, the latter information-theoretic quantities represent powerful instruments in quantifying causality between time-evolving systems. This is because ensuing information-theoretic functionals (typically based on Shannon entropy) quantify—in a non-parametric and explicitly non-symmetric way—the flow of information between two (or more) time series. In particular, transfer entropies (TEs) have recently received considerable attention. The catalyst was the infusion of new (numerical and conceptional) ideas. For instance, the performances of the Shannon entropy-based conditional entropies and conditional mutual entropies have been, in recent years, extensively tested using numerically-generated time series [17,22]. Sophisticated algorithms have been developed to uncover direct causal relations in multivariate time series [23,24,25]. In parallel, increasing attention has been devoted to the development of reliable estimators of entropic functionals to detect causality from nonlinear time series [26]. At the same time, it has been recognized that information-theoretic approaches play important roles in dealing with complex dynamical systems that are multiscale and/or non-Gaussian [21,27,28,29]. The latter class includes complex systems with heavy-tailed probability distributions epitomized, e.g., in financial and climatological time series [30,31].

In this paper, we extend the popular Shannon entropy-based TE (STE), which represents a prominent tool for assessing directed information flow between joint processes, and quantifies information transfer in terms of *Rényi’s TE* (RTE). RTE was introduced by one of us (PJ) in reference [21] in the context of a bivariate financial time series. The original idea was to use the RTE in order to exploit the theoretical formulation that could identify and quantify peculiar features in multiscale bivariate processes (e.g., multiscale patterns, generalized fractal dimensions, or multifractal cross-correlations) that are often seen in finance. In contrast to [21], where the focus was mostly on qualitative aspects of Rényian information flow between selected stock-market time series, in the present work, we wish to be more quantitative by analyzing coupled time series that are numerically generated from known dynamics. Specifically, we demonstrate how *the RTE method performs in the detection of the coupling direction and onset of synchronization between two Rössler oscillators* [32] *that are unidirectionally coupled in the first variable x*. The Rössler system (RS) is a paradigmatic and well-studied low-dimensional chaotic dynamical system. When coupled, RSs allow for *synchronization* as well as a subtle phenomenon known as “phase synchronization”, i.e., when the amplitudes of both systems are not correlated while the phases are approximately equal. In this respect, the synthetic bivariate time series (generated from coupled RSs) serves as an excellent test-bed, allowing to numerically analyze, e.g., drive–response relationships or identify the ensuing onset (or threshold) of synchronization. In doing so, we identify factors and influences that can lead to either decreases in the RTE sensitivity or false detections and propose some ways to cope with them. The aforementioned issues have not been explicitly studied in the framework of the RTE; this work presents the first attempt in this direction.

To set the stage, we shall first, in Section 2, provide the information-theoretic background on Rényi entropy (RE), which will be needed in the main body of the text. For self-consistency of our exposition, we briefly review Shannon’s transfer entropy of Schreiber and motivate and derive the core quantity of this work—the Rényi transfer entropy. The issue of causality (and its connection to RTE) is examined in Section 3. In particular, we prove that the Granger causality is entirely equivalent to the RTE for Gaussian processes and show how the Granger causality and the RTE are related in the case of heavy-tailed (namely α-Gaussian) processes. Section 4 is dedicated to derived information-theoretic concepts, such as the balance of transfer entropy and effective transfer entropy that will be employed in our analysis. The proposed framework is then illustrated on two unidirectionally coupled Rössler systems as a paradigmatic example. To cultivate our intuition about the latter RSs, we discuss in Section 5 the inner workings of such RSs in terms of simple numerical experiments. The ensuing numerical analysis is presented in Section 6, where we discuss how the RTE can be used to detect causality and the onset of synchronization in the two coupled RSs. We also demonstrate how the RTE provides non-trivial insight into the structure of a transient regime that exists between the regions of chaotic correlations and the onset of synchronization. Finally, Section 7 summarizes our theoretical and numerical findings and discusses possible extensions of the present work. For the reader’s convenience, we relegate some technical issues concerning the RE estimator employed and the statistical significance of results presented to Appendix A and Appendix B.

## 2. Rényi Entropy

Information theory approaches based on Shannon entropy currently belong in the portfolio of techniques and tools that are indispensable in addressing causality issues in complex dynamical systems. At the same time, Shannon’s information theory is limited in its scope. In fact, since Shannon’s seminal papers [33], it has been known that Shannon’s information measure (or entropy) represents mere idealized information, appearing only in situations when the buffer memory (or storage capacity) of a transmitting channel is infinite. In particular, Shannon’s source coding theorem (or noiseless coding theorem), which establishes the limits to possible data compression and, thus, provides operational meaning to the Shannon entropy, assumes that the *cost* of a codeword is a linear function of its length (so the optimal code has a minimal cost out of all codes). However, the linear costs of codewords are not always desirable. For instance, when the storage capacity is finite one would aim to penalize excessively lengthy codewords with a price that is, e.g., exponential rather than the linear function of the length.

For these reasons, information theorists have devised various remedies to deal with such cases. This usually consists of substituting Shannon’s information measure with information measures of other types. Consequently, numerous generalizations of Shannon’s entropy have started to proliferate in the information-theory literature, ranging from additive entropies [34,35] to a rich class of non-additive entropies [36,37,38,39,40], to more exotic types of entropies [41]. The one-parametric class of information measures, known as *Rényi entropies*, introduced by Hungarian mathematician and information theorist Alfred Rényi in the early 1960s [42,43], is particularly prominent among such generalizations. Applications of RE in information theory, namely its generalization to coding theorems, were carried over by Campbel [44], Csiszár [45,46], Aczél [47], and others. In a physical setting, RE was popularized in the context of chaotic dynamical systems by Kadanoff et al. [48] and in connection with multifractals by Mandelbrot [49]. RE is also indispensable in the quantum information theory where it quantifies multipartite entanglement [50].

In its essence, REs constitute a one-parametric family of information measures labeled by parameter α, fulfilling the additivity with respect to the composition of statistically independent systems. The special case with α=1 corresponds to ordinary Shannon’s entropy. REs belong to a broader class of so-called Uffink entropic functionals [51,52], i.e., the most general class of solutions that satisfy Shorem–Johnson axioms for the maximum entropy principle in the statistical estimation theory. Moreover, it might be shown that Rényi entropies belong to the class of the so-called mixing homomorphic functions [53] and that they are analytic for $\alpha \in C_{I\cup IV}$, cf. [34].

### 2.1. Definition

RE is defined as an exponentially weighted mean of the *Hartley information measure* −logp (i.e., elementary measure of information) [54]. In fact, it was shown by Rényi that, except for a linearly-weighted average (which leads to Shannon entropy), exponential weighting is the only possible averaging that is both compatible with the Kolmogorov–Nagumo average prescription and leads to entropies that are additive, with respect to independent systems [42,43]. RE, associated with a system described with a probability distribution P, reads

(1) $$H_{\alpha }\left[P\right]\;=\;\frac{1}{1-\alpha }\log_{2}\sum_{i=1}^{n}p_{i}^{\alpha }\;.$$

RE has the following properties [34,43]:

- RE is symmetric, i.e., $H_{\alpha }\left[\left\{p_{1},\cdots ,p_{n}\right\}\right]=H_{\alpha }\left[\left\{p_{\pi (1)},\cdots ,p_{\pi (n)}\right\}\right]$;
- RE is non-negative, i.e., $H_{\alpha }\geq 0$;
- $\lim_{\alpha \to 1}H_{\alpha }=H_{1}$, where $H_{1}=H$ is the Shannon entropy;
- $H_{0}=\log_{2}n$ is the *Hartley* entropy and $H_{2}=-\log_{2}\sum_{i=1}^{n}p_{i}^{2}$ is the *Collision entropy*;
- $0\leq H_{\alpha }\left[P\right]\leq \log_{2}n$;
- $H_{\alpha }$ is a positive, decreasing the function of α≥0.

Let us mention that $H_{\alpha }\left[P\right]$ with different αs complement each other. This is because for each specific α, the ensuing $H_{\alpha }\left[P\right]$ carries extra information that is not present in any other $H_{\beta }\left[P\right]$ with β≠α. In information theory, this fact is known as the *reconstruction theorem*, namely, the underlying distribution P can be uniquely reconstructed only if all $H_{\alpha }\left[P\right]$ are known, [21,34,55]. In chaotic dynamical systems, the reconstruction theorem goes under the name *complementary generalized dimensions* [56] (cf. also next subsection).

### 2.2. Multifractals, Chaotic Systems, and Rényi Entropy

Another appealing property of the Rényi entropy is its close connection to *multifractals*, i.e., the mathematical paradigm that is often encountered in complex dynamical systems with examples ranging from turbulence and strange attractors to meteorology and finance, see, e.g., [57]. The aforementioned connection is established through the so-called *generalized dimensions*, which are defined as [2,48]

(2) $$D_{\alpha }\;=\;-\lim_{\delta \to 0}\frac{H_{\alpha }\left(\delta \right)}{\log\delta }\;$$

where δ is a size of a δ−mesh covering of a configuration space of a system. Generalized dimensions $D_{\alpha }$ are conjugate to the *multifractal spectrum* f(β) through the Legendre transform [48]

(3) $$\left(\alpha -1\right)D_{\alpha }\;=\;\alpha \beta \;-\;f\left(\beta \right).$$

The function f(β) is called the multifractal spectrum because β plays the role of the scaling exponent in the local probability distribution, e.g., distribution with support on the *i*-th hypercube of a mesh size δ scale, as $p_{i}\left(\delta \right)∼\delta^{\beta_{i}}$. The key assumption in the multifractal analysis is that in the small δ− limit, the local probability distribution depends smoothly on β. It can be argued that f(β) corresponds to the (box-counting) fractal dimension of the portion of the configuration space where local probability distributions have the scaling exponent β, cf., e.g., reference [34]. In this way, the multifractal can be viewed as an ensemble of intertwined (uni)fractals, each with its own fractal dimension f(β).

The multifractal paradigm is particularly pertinent in the *theory of chaotic systems*. For instance, chaotic dynamics and strange attractors, in particular, are uniquely characterized by the infinite sequences of generalized dimensions $D_{\alpha }$, cf. reference [56]. In particular, the generalized dimensions can help to recognize (in a quantitative way) the main geometric features of chaotic systems. For instance, they may help to distinguish chaotic behavior from noisy behavior, determine the number of variables that are needed to model the dynamics of the system or classify systems into universality classes. On the other hand, dynamical features of chaotic systems are often analyzed through such quantifiers as *Lyapunov exponent*, which is a measure of the divergence of nearby trajectories, or ensuing *Kolmogorov-Sinai entropy rate* (KSE), which quantifies the change of entropy as the system evolves and is given by the sum of all positive Lyapunov exponents. The connection between KSE and the time evolution of the information-theoretic or statistical entropy is quite delicate, see, e.g., the discussion in reference [58], though the upshot is clear, in order to describe the dynamics of a (complex) system, the temporal change or the difference in entropy is more relevant than the entropy itself. Consequently, while RE (alongside with $D_{\alpha }$) is a suitable quantifier of geometric properties of chaotic systems, its temporal differences or temporal rates are useful for the description of the dynamics of such systems. Rényi’s transfer entropy follows the latter route.

### 2.3. Shannon Transfer Entropy

In order to understand the concept of Rényi transfer entropy, we recall first its Shannon’s counterpart.

Let $X={\left\{x_{i}\right\}}_{i=1}^{N}$ be a discrete random variable with ensuing probability distribution $P_{X}$, then the Shannon entropy of this process is

(4) $$H\left(X\right)\;\equiv \;H\left(P_{X}\right)\;=\;-\sum_{x\in X}p\left(x\right)\log_{2}p\left(x\right)\;.$$

Let $Y={\left\{y_{i}\right\}}_{i=1}^{N}$ be another random variable, then *mutual information* between *X* and *Y* is

(5) $$\begin{array}{ccc} I(X\;:\;Y)\; & = & \;\sum_{x\in X,\;\;y\in Y}p\left(x,y\right)\log_{2}\frac{p(x,y)}{p(x)p(y)}\\ & = & \;H(X)\;-\;H(X|Y)\;=\;H(Y)\;-\;H(Y|X)\;, \end{array}$$

where quantity H(X|Y) is the *conditional entropy*, defined as

(6) $$H\left(X|Y\right)\;=\;-\sum_{x\in X,\;\;y\in Y}p\left(x,y\right)\log_{2}p\left(x|y\right)\;.$$

Mutual information quantifies an average reduction in uncertainty (i.e., gain in information) about *X* resulting from the observation of *Y*, or vice versa. Since I(X:Y)=I(Y:X), it cannot be used as a measure of directional information flow. Note also that the amount of information contained in *X* about itself is just the Shannon entropy, i.e., I(X:X)=H(X).

The mutual information between two processes *X* and *Y* conditioned on the third process *Z* is called *conditional mutual information* and is defined as

(7) I(X:Y|Z)=H(X|Z)−H(X|Y,Z)=I(X:(Y,Z))−I(X:Y).

Let us now consider two time sequences (e.g., two stock market time series) described by stochastic (possibly vector-type) random variables $X_{t}$ and $Y_{t}$. Let us assume further that the time steps (e.g., data ticks) are discrete with the time step τ and with $t_{n}=t_{0}+n\tau$ where $t_{0}$ is some reference time. For practical purposes, it is also useful to assume that $X_{t}$ and $Y_{t}$ represent discrete-time stochastic Markov processes of order *k* and *l*, respectively.

We wish to know what information will be gained on $X_{t_{n+1}}$ by observing $Y_{t}$ up to time $t_{n}$. To this end, we introduce the joint process $X_{t_{n}},X_{t_{n-1}},\ldots ,X_{t_{n-k+1}}$, which we denote as $X_{n}^{\left(k\right)}$, and similarly, we define the joint process $Y_{n}^{\left(l\right)}\equiv Y_{t_{n}},Y_{t_{n-1}},\ldots ,Y_{t_{n-l+1}}$. By replacing *X* in (7) by $X_{t_{n+1}}$, *Y* by $Y_{n}^{\left(l\right)}$, and *Z* by $X_{n}^{\left(k\right)}$, we obtain the desired conditional mutual information

(8) $$\begin{array}{ccc} \;I(X_{t_{n+1}}:Y_{n}^{\left(l\right)}|X_{n}^{\left(k\right)})\;\; & = & \;\;H\left(X_{t_{n+1}}|X_{n}^{\left(k\right)}\right)\;-\;H\left(X_{t_{n+1}}|Y_{n}^{\left(l\right)},X_{n}^{\left(k\right)}\right)\\ & = & \;\;\sum_{x_{n}^{\left(k\right)}\in X_{n+1}^{\left(k\right)},\;\;y_{n}^{\left(l\right)}\in Y_{n}^{\left(l\right)}}p\left(x_{n+1},x_{n}^{\left(k\right)},y_{n}^{\left(l\right)}\right)\log_{2}\left(\frac{p(x_{n+1}|x_{n}^{\left(k\right)},y_{n}^{\left(l\right)})}{p(x_{n+1}|x_{n}^{\left(k\right)})}\right)\;. \end{array}$$

The conditional mutual information (8) is also known as *Shannon transfer entropy* from $Y_{t}$ to $X_{t}$ (or simply from *Y* to *X*) and as a measure of the directed (time asymmetric) information transfer between joint processes, it was introduced by Schreiber in reference [19]. The latter is typically denoted as

(9) $$T_{Y\to X}\left(k,l\right)\;\equiv \;I\left(X_{t_{n+1}}:Y_{n}^{\left(l\right)}|X_{n}^{\left(k\right)}\right)\;.$$

As already mentioned, for independent processes, TE is equal to zero. For a non-zero case transfer, entropy measures the deviation from the independence of the two processes. An important property of the transfer entropy is that it is directional, i.e., in general, $T_{Y\to X}\neq T_{X\to Y}$.

### 2.4. Rényi Transfer Entropy

In the same manner as in (7), we can introduce the *Rényi transfer entropy of order α* from *Y* to *X* (see also reference [21]) as

(10) $$\begin{array}{ccc} T_{\alpha ,Y\to X}^{R}\left(k,l\right)\; & = & \;H_{\alpha }\left(X_{t_{n+1}}|X_{n}^{\left(k\right)}\right)\;-\;H_{\alpha }\left(X_{t_{n+1}}|X_{n}^{\left(k\right)},Y_{n}^{\left(l\right)}\right)\\ & = & \;I_{\alpha }\left(X_{t_{n+1}}:Y_{n}^{\left(l\right)}|X_{n}^{\left(k\right)}\right)\;, \end{array}$$

where $H_{\alpha }\left(X|Y\right)$ is the *conditional entropy of order α* and $I_{\alpha }\left(X:Y\right)$ is the *mutual information of order α*. These can be explicitly written as [21,43]

(11) $$\begin{array}{ccc} H_{\alpha }\left(X|Y\right)\; & = & \;\frac{1}{1-\alpha }\log_{2}\frac{\sum_{x\in X,y\in Y}p^{\alpha }\left(x,y\right)}{\sum_{y\in Y}p^{\alpha }\left(y\right)}\;,\\ I_{\alpha }\left(X:Y\right)\; & = & \;\frac{1}{1-\alpha }\log_{2}\frac{\sum_{x\in X,y\in Y}p^{\alpha }\left(x\right)p^{\alpha }\left(y\right)}{\sum_{x\in X,y\in Y}p^{\alpha }\left(x,y\right)}\;. \end{array}$$

It can be checked (via L’Hospital’s rule) that Rényi’s transfer α-entropy reduces to Shannon TE in the α→1 limit, i.e.,

(12) $$\begin{array}{c} \lim_{\alpha \to 1}T_{\alpha ,Y\to X}^{R}\;=\;T_{Y\to X}\;. \end{array}$$

From (10), we see that $T_{\alpha ,Y\to X}^{R}\left(k,l\right)$ may be intuitively interpreted as the degree of ignorance (or uncertainty) about $X_{t_{n+1}}$ resolved by the past states $Y_{n}^{\left(l\right)}$ and $X_{n}^{\left(k\right)}$, over and above the degree of ignorance about $X_{t_{n+1}}$ already resolved by its own past state alone. Here, the ignorance is quantified by the Rényi information measure (i.e., RE) of order α.

Rényi TE can also be negative (unlike the Shannon TE). This means that the uncertainty of the process $X_{t}$ becomes bigger knowing the past of $Y_{t}$, i.e., $H_{\alpha }\left(X_{t_{n+1}}|X_{n}^{\left(k\right)}\right)\leq H_{\alpha }\left(X_{t_{n+1}}|X_{n}^{\left(k\right)},Y_{n}^{\left(l\right)}\right)$. If $X_{t}$ and $Y_{t}$ are independent, then $T_{\alpha ,Y\to X}^{R}=T_{\alpha ,X\to Y}^{R}=0$. However, in contrast to Shannon’s case, the fact that $T_{\alpha ,Y\to X}^{R}=0$ does necessarily imply the independence of the two underlying stochastic processes. Nonetheless, in Section 3, we prove that in case of Gaussian (Wiener) processes, 0-valued RTE is a clear signature of independence.

Due to the *reconstruction theorem* mentioned in Section 2.1, RTE $T_{\alpha ,Y\to X}^{R}$ conveys for each α a different type of directional information from *Y* to *X*. The essence of this statement can be understood qualitatively by introducing the so-called *escort distribution*.

### 2.5. Escort Distribution

Because of the nonlinear way in which probability distributions enter in the definition of RE, cf. Equation (1), the RTE represents a useful measure of transmitted information that quantifies the dominant information flow between certain parts of underlying distributions. In fact, for 0<α<1, the corresponding information flow accentuates marginal events, while for α>1, more probable (close-to-average) events are emphasized [21]. In this respect, one can zoom or amplify different parts of probability density functions involved by merely choosing appropriate values of α. This is particularly useful in studies of time sequences, where marginal events are of crucial importance, for instance, in financial time series.

In order to better understand the aforementioned “zooming” property of RTE, we rewrite (10) in the form

(13) $$T_{\alpha ,Y\to X}^{R}\left(k,l\right)\;=\;\frac{1}{1-\alpha }\log_{2}\left(\frac{\sum \frac{p^{\alpha }\left(x_{n}^{\left(k\right)}\right)}{\sum p^{\alpha }\left(x_{n}^{\left(k\right)}\right)}p^{\alpha }\left(x_{n+1}|x_{n}^{\left(k\right)}\right)}{\sum \frac{p^{\alpha }\left(x_{n}^{\left(k\right)},y_{n}^{\left(l\right)}\right)}{\sum p^{\alpha }\left(x_{n}^{\left(k\right)},y_{n}^{\left(l\right)}\right)}p^{\alpha }\left(x_{n+1}|x_{n}^{\left(k\right)},y_{n}^{\left(l\right)}\right)}\right)\;.$$

**Figure 1:**
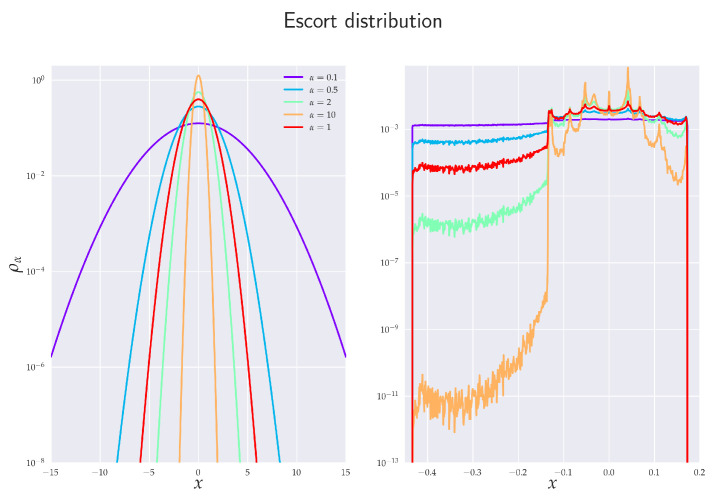
Illustration of the concept of escort distribution $\rho_{\alpha }$ on histograms. The left figure depicts log-scaled normal distribution N(0,1), while in the right figure, we show the log-scaled histogram for $x_{1}-$projection increments from the Rössler system (52). Both figures demonstrate that the escort distribution deforms the original distribution (α=1) so that 0<α<1 less probable events are emphasized (the smaller, α the greater emphasis) while high probable events are accordingly suppressed. For α>1, the situation is reversed.

This particular representation shows how the underlying distribution changes (or deforms) with the change of parameter α. The numerator and denominator inside the log-function contain the so-called *escort* (or *zooming*) *distributions* $\rho_{\alpha }$

(14) $$\rho_{\alpha }\left(x\right)\;\equiv \;\frac{p^{\alpha }\left(x\right)}{\sum_{x\in X}p^{\alpha }\left(x\right)}\;,$$

which emphasize less probable events for 0<α<1 and more probable events when α>1, see Figure 1.

Note also that $\rho_{\alpha }\left(x_{n}^{\left(k\right)},y_{n}^{\left(l\right)}\right)$ is not the joint probability distribution of $X_{n}^{\left(k\right)}$ and $Y_{n}^{\left(l\right)}$ as it does not satisfy the Kolmogorov–de Finetti relation for conditional probabilities [59].

In connection with (13), we may note that for 0<α<1 the multiplicative factor is positive, and so the RTE is negative if, by learning $Y_{n}^{\left(l\right)}$, the rare events are (on average) more emphasized than in the case when only $X_{n}^{\left(k\right)}$ alone is known. Analogically, for α>1 the RTE can be negative when—by learning $Y_{n}^{\left(l\right)}$—the more probable events are (on average) more accentuated in comparison with the situation when $Y_{n}^{\left(l\right)}$ is not known. It should be stressed that the analogous situation does not hold for Shannon’s TE. This is because in the limit α→1 we regain expression (8), which is nothing but relative entropy, and as such, it is always non-negative due to Gibbs inequality. At the same time, Shannon’s TE is, by its very definition, also mutual information. While RTE is also defined to be a mutual information, it is not relative entropy (in the RE case, those two concepts do not coincide). It can be shown (basically via Jensen’s inequality) [34] that the relative entropy based on RE is also non-negative but this is not true for ensuing mutual information, which serves as a conceptual basis for the definition of RTE.

## 3. Rényi Transfer Entropy and Causality

As already seen, Rényi TE (analogously to Shannon TE) is a directional measure of information transfer. Let us now comment on the connection of the RTE with the causality concept.

### 3.1. Granger Causality—Gaussian Variables

The first general definition of causality, which could be quantified and measured computationally was given by Wiener in 1956, namely “*…For two simultaneously measured signals, if we can predict the first signal better by using the past information from the second one than by using the information without it, then we call the second signal causal to the first one…*” [9].

The introduction of the concept of causality into the experimental practice, namely into analyses of data observed in consecutive time instants (i.e., time series), is due to the Nobel prize winner (economy, 2003) C.W.J. Granger. The so-called *Granger causality* is defined so that the process $Y_{t}$ *Granger causes* another process $X_{t}$ if, in an appropriate statistical sense, $Y_{t}$ assists in predicting the future of $X_{t}$ beyond the degree to which $X_{t}$ already predicts its own future.

The standard test of the Granger causality was developed by Granger himself [10] and it is based on a linear regression model, namely

(15) $$\begin{array}{c} X_{t}\;=\;a_{0t}\;+\;\sum_{\ell =1}^{k}a_{1\ell }X_{t-\ell }\;+\;\sum_{\ell =1}^{l}a_{2\ell }Y_{t-\ell }\;+\;e_{t}\;, \end{array}$$

where $a_{0},a_{1\ell },a_{2\ell }$ are (constant) regression coefficients, *l* and *k* represent the maximum number of lagged observations included in the model (i.e., memory indices), *t* is a discrete time with the time step τ (*ℓ* is also quantified in units of τ) and $e_{t}$ is the uncorrelated random variable (residual) with zero mean and variance $\sigma^{2}$. The *null hypothesis* that $Y_{t}$ does not cause $X_{t}$ (in the sense of Granger) is not rejected if and only if $a_{2\ell }=0$ for ℓ=1,…,l. In the latter case, we will call the ensuing regression model the *reduced regression model*.

It is not difficult to show that for Gaussian variables, the RTE and Granger causality are entirely equivalent. To see this, we use the *standard measure* of the Granger causality, which is defined as [60]

(16) $$\begin{array}{c} F_{Y\to X}^{\left(k,l\right)}\;=\;\log_{2}\frac{\left|\sum (e_{t}^{\prime })\right|}{\left|\sum (e_{t})\right|}\;, \end{array}$$

where ∑(…) is the covariance matrix, |…| denotes the matrix determinant, and $e_{t}$, $e_{t}^{\prime }$ are residuals in the full and reduced regression model, respectively. We chose the logarithm to the base 2, rather than *e* for technical convenience. We now prove the following theorem:

**Theorem** **1.** *If the joint process $X_{t}$, $Y_{t}$ is Gaussian, then there is an exact equivalence between the Granger causality and RTE, namely*

(17) $$\begin{array}{c} F_{Y\to X}^{\left(k,l\right)}\;=\;2T_{\alpha ,Y\to X}^{R}\left(k,l\right)\;. \end{array}$$

This can be proved in the following way (for an analogous proof for Shannon’s TE, see [61]). We first define the *partial covariance* as

(18) $$\begin{array}{c} \sum \left(X|Y\right)\;=\;\Sigma \left(X\right)\;-\;\Sigma \left(X,Y\right)\Sigma {\left(Y\right)}^{-1}\Sigma {\left(X,Y\right)}^{⊤}\;, \end{array}$$

where ${\sum (X)}_{ij}=\mathrm{cov}\left(X_{i},X_{j}\right)$ and ${\sum (X,Y)}_{ij}=\mathrm{cov}\left(X_{i},Y_{j}\right)$ with X and Y being random vector (or multivariate) variables. Let X and Y be jointly distributed random vectors in the linear regression model

(19) $$\begin{array}{c} X\;=\;a\;+\;YA\;+\;e\;. \end{array}$$

Here, a is a constant vector, A contains regression coefficients, and **e** is a residual random vector with zero mean. In the subsequent, we will identify both X and Y with stochastic vectors (see text after Equation (28)). In such a case, one can always choose a specified number of time lags, so that system (19) (or better (23) and, consequently, (22)) is uniquely solvable, as neither vector a nor matrix A are time-dependent.

We now apply the least square method to the mean square error

(20) $$\begin{array}{c} {ℰ}^{2}\;\equiv \;\sum_{i}E\left(e_{i}^{2}\right)\;=\;\sum_{i}E\left[{\left(X-YA-a\right)}_{i}^{2}\right]\;, \end{array}$$

Here, E(…) denotes the average value. The ensuing least square equations

(21) $$\begin{array}{c} \frac{\partial {ℰ}^{2}}{\partial A_{ij}}\;=\;0\;\;\;\;and\;\;\;\;\frac{\partial {ℰ}^{2}}{\partial a_{k}}\;=\;0\;, \end{array}$$

yield

(22) $$\begin{array}{ccc} & & a_{l}\;=\;E\left(X_{l}\right)\;-\;\sum_{k}E\left(Y_{k}\right)A_{kl}\;, \end{array}$$

(23) $$\begin{array}{ccc} & & A_{li}\;=\;\sum_{j}{\left[\sum \left(X\right)\right]}_{lj}^{-1}\;\;\sum {\left(Y,X\right)}_{ji}\;. \end{array}$$

From (19) follows that

(24) $$\begin{array}{c} E\left(X_{i}X_{j}\right)\;=\;E\left[{\left(a\;+\;YA\;+\;e\right)}_{i}{\left(a\;+\;YA\;+\;e\right)}_{j}\right]\;, \end{array}$$

which after employing (22) can be equivalently rewritten as

(25) $$\begin{array}{c} \mathrm{cov}\left(X_{i},X_{j}\right)\;=\;\sum_{l,k}\mathrm{cov}\left(Y_{l},Y_{k}\right)A_{li}A_{kj}\;+\;\mathrm{cov}\left(e_{i},e_{j}\right)\;, \end{array}$$

or equivalently

(26) $$\begin{array}{c} \sum \left(X\right)\;=\;A^{⊤}\sum \left(Y\right)A\;+\;\sum \left(e\right)\;. \end{array}$$

If we now insert (23)–(26), we obtain

(27) $$\begin{array}{c} \mathrm{cov}\left(e_{i},e_{j}\right)\;=\;\mathrm{cov}\left(X_{i},X_{j}\right)\;-\;\mathrm{cov}\left(X_{i},Y_{k}\right){\left[\mathrm{cov}\left(Y_{k},Y_{i}\right)\right]}^{-1}{\left[\mathrm{cov}\left(X_{i},Y_{j}\right)\right]}^{⊤}\;, \end{array}$$

which might be equivalently written as

(28) $$\begin{array}{c} \sum (e)\;=\;\sum (X|Y)\;. \end{array}$$

If we now take $X=(X_{t_{n+1}})$, $a=(a_{0})$, $Y=(X^{\left(k\right)},Y^{\left(l\right)})$, $A=\mathrm{diag}(a_{1n}^{\left(k\right)},a_{2n}^{\left(l\right)})$ for the full regression model and $Y=(X_{n}^{\left(k\right)})$, $A=\mathrm{diag}(a_{1}^{\left(k\right)})$ for the reduced regression model, we might write that

(29) $$\begin{array}{c} F_{Y\to X}^{\left(k,l\right)}\;=\;\log_{2}\frac{\left|\sum (e_{t}^{\prime })\right|}{\left|\sum (e_{t})\right|}\;=\;\log_{2}\left(\frac{\left|\sum (X_{t_{n+1}}|X_{n}^{\left(k\right)})\right|}{\left|\sum (X_{t_{n+1}}|X_{n}^{\left(k\right)},Y_{n}^{\left(l\right)})\right|}\right)\;. \end{array}$$

At this stage, we can use the fact that RE of the multivariate Gaussian variable X is [62]

(30) $$\begin{array}{c} H_{\alpha }\left(X\right)\;=\;\frac{1}{2}\log_{2}\left|\sum \left(X\right)\right|\;+\;\frac{D_{{}_{X}}}{2}\log_{2}\left(2\pi \alpha^{\alpha^{\prime }/\alpha }\right)\;. \end{array}$$

Here, $D_{{}_{X}}$ is the dimension of X and $\alpha^{\prime }$ is a Hölder dual variable to α (i.e., $1/\alpha +1/\alpha^{\prime }=1$). In particular, for jointly multivariate Gaussian variables X and Y, we can use (11) to write

(31) $$\begin{array}{ccc} H_{\alpha }\left(X|Y\right) & = & \left[\frac{1}{2}\log_{2}\left|\sum \left(X⊕Y\right)\right|+\frac{D_{{}_{X}}+D_{{}_{Y}}}{2}\log_{2}\left(2\pi \alpha^{\alpha^{\prime }/\alpha }\right)\right]\\ & - & \left[\frac{1}{2}\log_{2}\left|\sum \left(Y\right)\right|+\frac{D_{{}_{Y}}}{2}\log_{2}\left(2\pi \alpha^{\alpha^{\prime }/\alpha }\right)\right]\\ & = & \frac{1}{2}\log_{2}\left|\sum \left(X|Y\right)\right|\;+\;\frac{D_{{}_{X}}}{2}\log_{2}\left(2\pi \alpha^{\alpha^{\prime }/\alpha }\right)\;. \end{array}$$

Here, ⊕ denotes the direct sum. Employing finally the defining relation (10), we obtain

(32) $$\begin{array}{ccc} T_{\alpha ,Y\to X}^{R}\left(k,l\right)\; & = & \;H_{\alpha }\left(X_{t_{n+1}}|X_{n}^{\left(k\right)}\right)\;-\;H_{\alpha }\left(X_{t_{n+1}}|X_{n}^{\left(k\right)},Y_{n}^{\left(l\right)}\right)\\ & = & \frac{1}{2}\log_{2}\left(\frac{\left|\sum (X_{t_{n+1}}|X_{n}^{\left(k\right)})\right|}{\left|\sum (X_{t_{n+1}}|X_{n}^{\left(k\right)},Y_{n}^{\left(l\right)})\right|}\right)\;. \end{array}$$

This confirms the statement of Theorem 1. In addition, since the standard measure of Granger causality (16) is typically defined only for the univariate target and source variables $X_{t}$ and $Y_{t}$, we can omit |…| in (29) and (32).

Theorem 1 deserves two comments. First, the theorem is clearly true for any α. In fact, it is α independent, which means that for Gaussian processes we can employ any RTE to test the Granger causality. This naturally generalizes the classical result of Barnett et al. [61] (see also [1]) that is valid for Shannon’s TE. When TE is phrased in terms of the Shannon entropy, it is typically easier to use various multivariate autoregressive model fitting techniques (e.g., the Lewinson–Wiggins–Robinson algorithm or the least-squares linear regression approach [63]) to derive $F_{Y\to X}^{\left(k,l\right)}$ more efficiently than by employing direct entropy/mutual information-based estimators. On the other hand, since the efficiency and robustness of RTE estimators crucially hinge on the parameter α employed [64] (see also our discussion in Section 4), it might be, in many cases, easier to follow the information-theoretic route to the Granger causality (provided the Gaussian framework is justified). One can even test the Gaussian assumption in the actual time series by determining the RTE for various α parameters and checking if the results are α independent.

Second, the exact equivalence between the Granger causality and RTE can be (in the Gaussian case) retraced to the fact that in Equation (30) the second additive term on the RHS is proportional to $D_{X}$. It is not difficult to see (by a direct inspection) that this proportionality will be preserved in many other exponential distributions that satisfy the Markov factorization property. In these cases, the equivalence between the Granger causality and RTE statistics will also be preserved. However, for generic distributions, the additive term in (30) will no longer be a linear function of $D_{X}$ and, hence, it will not be canceled. This, in turn, spoils the desired equivalence. In the following section, we will discuss one possible generalization of Theorem 1 in the context of heavy-tailed distributions.

### 3.2. Granger Causality—Heavy-Tailed Variables

It is not difficult to find relations analogous to (32) in a more general setting. Here, we will illustrate this point with heavy-tailed (namely α-Gaussian) random variables, where computations can be conducted analytically.

**Figure 2:**
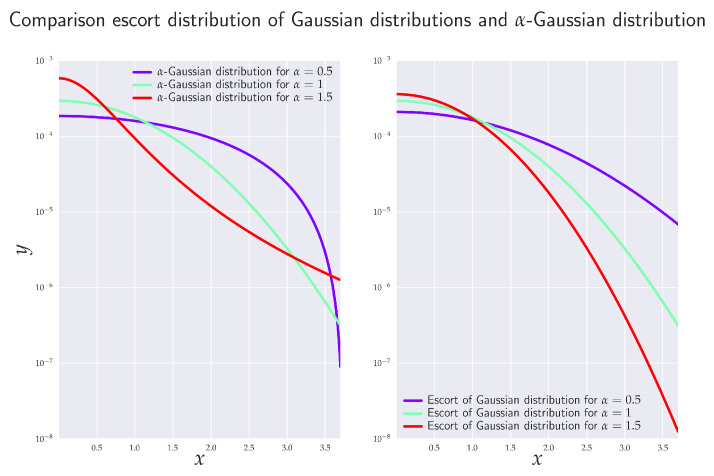
Comparison of the escort distributions $\rho_{\alpha }$ of the Gaussian (normal) distribution N(0,1) and α-Gaussian distributions (in log-linear plots) with a choice of β in (33), such that variances are the same for equal αs. For α=1, the two distributions correspond to the Gaussian distribution N(0,1). Even though $\rho_{\alpha }$ and α-Gaussian distributions deform the same underlying Gaussian distribution N(0,1), α-Gaussian is (save for α=1) heavy-tailed, while $\rho_{\alpha }$ remains Gaussian.

It is well known that if variance and mean are the only statistical observables, then the conventional maximum entropy principle (MaxEnt) based on Shannon entropy yields Gaussian distribution. Similarly, if the very same MaxEnt is applied to Rényi entropy $H_{\alpha }$, one obtains the so-called α-Gaussian distribution [34] (cf. also Figure 2)

(33) $$\begin{array}{c} p_{i}\;=\;\frac{1}{Z_{\alpha }}{\left[1-\beta \left(\alpha -1\right)x_{i}^{2}\right]}_{+}^{1/(\alpha -1)}\;, \end{array}$$

that decays asymptotically following power law. Here, $\beta \in R^{+}$ and ${\left[z\right]}_{+}=z$ if z≥0 and 0, otherwise, $Z_{\alpha }$ is the normalization factor. It is more conventional to write (33) as

(34) $$\begin{array}{c} p_{i}\;=\;Z_{\alpha }^{-1}\;\;\exp_{\left\{2-\alpha \right\}}\left(-\beta x_{i}^{2}\right)\;, \end{array}$$

where

(35) $$\begin{array}{c} e_{\left\{\alpha \right\}}^{x}\;=\;{\left[1\;+\;(1-\alpha )x\right]}_{+}^{1/(1-\alpha )}\;, \end{array}$$

is the Box–Cox α-exponential [30].

α-Gaussian distribution (33) has finite variance (and, more generally, the covariance matrix) for $\frac{D}{2+D}<\alpha \leq 1$. Let us now assume that Granger’s linear (full/reduced) regression model is described by joint processes $X_{t}$ and $Y_{t}$ that are α-Gaussian. We now prove the following theorem:

**Theorem** **2.** *If the joint process $X_{t}$, $Y_{t}$ is α-Gaussian with $\alpha \in \left(\frac{1+k+l}{3+k+l},1\right]$ (i.e., a finite covariance matrix region) then $F_{Y\to X}^{\left(k,l\right)}-2T_{\alpha ,Y\to X}^{R}\left(k,l\right)$ is a monotonically decreasing function of α (at fixed k and l) with zero reached at a stationary point α=1. The leading-order correction to the Granger causality is “k”-independent and has the form*

(36) $$\begin{array}{c} F_{Y\to X}^{\left(k,l\right)}\;=\;2T_{\alpha ,Y\to X}^{R}\left(k,l\right)\;+\;\frac{l{\left(\alpha -1\right)}^{2}}{4}\;+\;O\left({\left(\alpha -1\right)}^{3}\right)\;. \end{array}$$

This result explicitly illustrates how certain “soft” heavy-tailed processes can be related to the concept of the Granger causality via universal types of corrections that are principally discernible in data analysis.

Theorem 2 can be proved in close analogy with our proof of Theorem 1. In fact, all steps in the proof are identical up to Equation (29). For the *D*-dimensional α-Gaussian process, the scaling property (30) reads

(37) $$\begin{array}{c} H_{\alpha }\left(X\right)\;=\;\frac{1}{2}\log_{2}\left|\sum \left(X\right)\right|\;+\;H_{\alpha }\left(Z_{\alpha }^{1,D}\right)\;. \end{array}$$

Here, $Z_{\alpha }^{1,D}$ represents an α-Gaussian random vector with zero mean and unit (D×D) covariance matrix. Relation (37) results from the following chain of identities

(38) $$\begin{array}{ccc} H_{\alpha }\left(X\right)\;\; & = & \;\;H_{\alpha }\left(\sqrt{\sum (X)}\;\;Z_{\alpha }^{1,D}\right)\\ & = & \;\;\frac{1}{1-\alpha }\log_{2}\int_{R^{D}}d^{D}y{\left(\int_{R^{D}}d^{D}z\;\delta \left(y-\sqrt{\sum (X)}\;\;z\right)F\left(z\right)\right)}^{\;\alpha }\\ & = & \;\;\frac{1}{1-\alpha }\log_{2}\left[{\left|\sum \left(X\right)\right|}^{(1-\alpha )/2}\int_{R^{D}}d^{D}y\;\;F^{\alpha }\left(y\right)\right]\\ & = & \;\;\frac{1}{2}\log_{2}\left|\sum \left(X\right)\right|\;+\;H_{\alpha }\left(Z_{\alpha }^{1,D}\right)\;, \end{array}$$

which is clearly valid for any non-singular covariance matrix. The derivation F(…) denoted the α-Gaussian probability density function with the unit covariance matrix and zero mean. We can now use the simple fact that

(39) $$\begin{array}{ccc} & & \;H_{\alpha }\left(Z_{\alpha }^{1,D}\right)=\log_{2}\left[{\left(\frac{\pi }{b(1-\alpha )}\right)}^{D/2}\;\;\frac{\Gamma \left(\frac{1}{1-\alpha }-\frac{D}{2}\right)}{\Gamma \left(\frac{1}{1-\alpha }\right)}\;\;{\left(1-\frac{D}{2\alpha }\left(1-\alpha \right)\right)}^{1/(\alpha -1)}\right]\\ & & \;=\frac{D}{2}\log_{2}\left[2\pi \alpha \right]\;+\;\log_{2}\left[\frac{\Gamma \left(\frac{1}{1-\alpha }-\frac{D}{2}\right)}{{\left(1-\alpha \right)}^{D/2}\Gamma \left(\frac{1}{1-\alpha }\right)}\right]\;+\;\log_{2}\left[{\left(1-\frac{D}{2\alpha }\left(1-\alpha \right)\right)}^{\frac{D}{2}-\frac{1}{1-\alpha }}\right], \end{array}$$

(where $b={\left[2\alpha -D\left(1-\alpha \right)\right]}^{-1}$), to write

(40) $$\begin{array}{ccc} H_{\alpha }\left(X|Y\right) & = & \frac{1}{2}\log_{2}\left|\sum \left(X|Y\right)\right|\;+\;H_{\alpha }\left(Z_{\alpha }^{1,D_{{}_{X}}+D_{{}_{Y}}}\right)\;-\;H_{\alpha }\left(Z_{\alpha }^{1,D_{{}_{Y}}}\right)\;. \end{array}$$

At this stage, we note that

(41) $$\begin{array}{ccc} H_{\alpha }\left(Z_{\alpha }^{1,D_{{}_{X}}+D_{{}_{Y}}}\right)\; & - & \;H_{\alpha }\left(Z_{\alpha }^{1,D_{{}_{Y}}}\right)\;-\;H_{\alpha }\left(Z_{\alpha }^{1,D_{{}_{X}}}\right)\\ & = & \;H_{\alpha }\left(Z_{\alpha }^{1,D_{{}_{X}}}|Z_{\alpha }^{1,D_{{}_{Y}}}\right)\;-\;H_{\alpha }\left(Z_{\alpha }^{1,D_{{}_{X}}}\right)\;, \end{array}$$

which is not zero as it was in the case of the Gaussian distribution. In fact, from the foregoing discussion, it is clear that for the α-Gaussian random variables, we can write the RTE in the form

(42) $$\begin{array}{ccc} & & \;T_{\alpha ,Y\to X}^{R}\left(k,l\right)\;=\;H_{\alpha }\left(X_{t_{n+1}}|X_{n}^{\left(k\right)}\right)\;-\;H_{\alpha }\left(X_{t_{n+1}}|X_{n}^{\left(k\right)},Y_{n}^{\left(l\right)}\right)\\ & & =\;\frac{1}{2}\log_{2}\left(\frac{\sum (X_{t_{n+1}}|X_{n}^{\left(k\right)})}{\sum (X_{t_{n+1}}|X_{n}^{\left(k\right)},Y_{n}^{\left(l\right)})}\right)\;+\;H_{\alpha }\left(Z_{\alpha }^{1,1}|Z_{\alpha }^{1,k}\right)\;-\;H_{\alpha }\left(Z_{\alpha }^{1,1}|Z_{\alpha }^{1,k+l}\right)\\ & & =\;\frac{1}{2}F_{Y\to X}^{\left(k,l\right)}\;+\;I_{\alpha }\left(Z_{\alpha }^{1,1}:Z_{\alpha }^{1,l}|Z_{\alpha }^{1,k}\right)\;. \end{array}$$

Here, we have set $Z_{\alpha }^{1,1}$ to correspond to the random variable $X_{t_{n+1}}$ with unit variance. Similarly, $Z_{\alpha }^{1,k}$ and $Z_{\alpha }^{1,l}$ correspond to unit covariance random variables $X_{n}^{\left(k\right)}$ and $Y_{n}^{\left(l\right)}$, respectively.

Clearly, when $Y_{t}$ and $X_{t}$ processes are independent (and, hence, *not causal* in the Granger sense), their joint distribution factorizes and, thus, $H_{\alpha }\left(Z_{\alpha }^{1,D_{{}_{X}}+D_{{}_{Y}}}\right)\mapsto H_{\alpha }\left(Z_{\alpha }^{1,D_{{}_{X}}}\times Z_{\alpha }^{1,D_{{}_{Y}}}\right)$. Additivity of the RE then ensures that $H_{\alpha }\left(Z_{\alpha }^{1,1}|Z_{\alpha }^{1,k}\right)\;=\;H_{\alpha }\left(Z_{\alpha }^{1,1}|Z_{\alpha }^{1,k+l}\right)$ and, hence, $I_{\alpha }\left(Z_{\alpha }^{1,1}:Z_{\alpha }^{1,l}|Z_{\alpha }^{1,k}\right)$ is zero. In other words, when two processes are not Granger causal, their RTEs are zero. Actually, it is not difficult to see that this is true irrespective of a specific form of the distribution involved. However, the opposite is not true since $I_{\alpha }\left(Z_{\alpha }^{1,1}:Z_{\alpha }^{1,l}|Z_{\alpha }^{1,k}\right)$ might be (unlike in Shannon’s case) negative; consequently, $T_{\alpha ,Y\to X}^{R}\left(k,l\right)$ can be zero even if $F_{Y\to X}^{\left(k,l\right)}$ is not. To understand this point better, we explicitly evaluate $I_{\alpha }\left(Z_{\alpha }^{1,1}:Z_{\alpha }^{1,l}|Z_{\alpha }^{1,k}\right)$ for our α-Gaussian random variables. Using (39), we can write

(43) $$\begin{array}{ccc} \;I_{\alpha }\left(Z_{\alpha }^{1,1}:Z_{\alpha }^{1,l}|Z_{\alpha }^{1,k}\right) & = & \log_{2}\left[\frac{\Gamma \left(\frac{1}{1-\alpha }-\frac{1+k}{2}\right)}{\Gamma \left(\frac{1}{1-\alpha }-\frac{k}{2}\right)}\frac{\Gamma \left(\frac{1}{1-\alpha }-\frac{k+l}{2}\right))}{\Gamma \left(\frac{1}{1-\alpha }-\frac{1+k+l}{2}\right)}\right]\\ & + & \log_{2}\left[\frac{{\left(\frac{\alpha }{1-\alpha }-\frac{1+k}{2}\right)}^{\frac{1+k}{2}-\frac{1}{1-\alpha }}}{{\left(\frac{\alpha }{1-\alpha }-\frac{k}{2}\right)}^{\frac{k}{2}-\frac{1}{1-\alpha }}}\frac{{\left(\frac{\alpha }{1-\alpha }-\frac{k+l}{2}\right)}^{\frac{k+l}{2}-\frac{1}{1-\alpha }}}{{\left(\frac{\alpha }{1-\alpha }-\frac{1+k+l}{2}\right)}^{\frac{1+k+l}{2}-\frac{1}{1-\alpha }}}\right]. \end{array}$$

**Figure 3:**
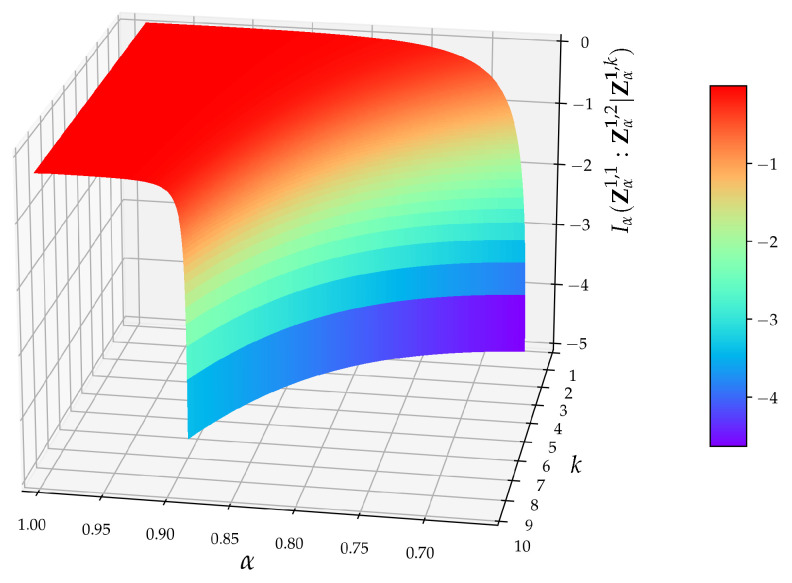
Example of $I_{\alpha }\left(Z_{\alpha }^{1,1}:Z_{\alpha }^{1,l}|Z_{\alpha }^{1,k}\right)$ for l=2 and k=1,2,…,10. Range validity of α is thus between $\frac{3+k}{5+k}$ and 1.

By setting $\zeta =\frac{1}{1-\alpha }-\frac{k}{2}$ and $\xi =\frac{1}{1-\alpha }-\frac{k+l}{2}$, we can rewrite (43) as

(44) $$\begin{array}{ccc} I_{\alpha }\left(Z_{\alpha }^{1,1}:Z_{\alpha }^{1,l}|Z_{\alpha }^{1,k}\right) & = & \log_{2}\left[\frac{\Gamma \left(\zeta -\frac{1}{2}\right)}{\Gamma \left(\zeta \right)}\frac{{\left(\zeta -1\right)}^{\zeta }}{{\left(\zeta -\frac{3}{2}\right)}^{\zeta -\frac{1}{2}}}\frac{\Gamma \left(\xi \right)}{\Gamma \left(\xi -\frac{1}{2}\right)}\frac{{\left(\xi -\frac{3}{2}\right)}^{\xi -\frac{1}{2}}}{{\left(\xi -1\right)}^{\xi }}\right]\\ & = & \log_{2}\left[\frac{\Gamma \left(\zeta -\frac{3}{2}\right)}{\Gamma \left(\zeta -1\right)}\frac{{\left(\zeta -1\right)}^{\zeta -1}}{{\left(\zeta -\frac{3}{2}\right)}^{\zeta -\frac{3}{2}}}\frac{\Gamma \left(\xi -1\right)}{\Gamma \left(\xi -\frac{3}{2}\right)}\frac{{\left(\xi -\frac{3}{2}\right)}^{\xi -\frac{3}{2}}}{{\left(\xi -1\right)}^{\xi -1}}\right]\\ & \leq & -\frac{1}{2}\log_{2}\left[\frac{\left(\xi -1\right)}{\left(\xi -\frac{3}{2}\right)}\right]\;\leq \;0\;, \end{array}$$

where on the last line we use the Kečkić–Vasić inequality [65]

(45) $$\begin{array}{c} \frac{{\left(x+1\right)}^{x+1}}{{\left(x+s\right)}^{x+s}}\;\;e^{s-1}\;\leq \;\frac{\Gamma (x+1)}{\Gamma (x+s)}\;\leq \;\frac{{\left(x+1\right)}^{x+\frac{1}{2}}}{{\left(x+s\right)}^{x+s-\frac{1}{2}}}\;\;e^{s-1}\;, \end{array}$$

valid for s∈(0,1). In addition, it can be numerically checked that $\frac{dI_{\alpha }\left(Z_{\alpha }^{1,1}:Z_{\alpha }^{1,l}|Z_{\alpha }^{1,k}\right)}{d\alpha }>0$, for all l,k from the definition, so the maximum of $I_{\alpha }\left(Z_{\alpha }^{1,1}:Z_{\alpha }^{1,l}|Z_{\alpha }^{1,k}\right)$ is attained at α=1, see Figure 3. When α is close to 1, then one can employ the asymptotic relation $\Gamma \left[x+\gamma \right]∼\Gamma \left[x\right]x^{\gamma }$ valid for x≫1, γ∈C, and rewrite (39) in the form $\left(D/2\right)\log_{2}\left[2\pi \alpha e^{\alpha }\right]$. In this case, (43) tends to zero and we obtain equivalence between TE and the Granger causality. This result should not be so surprising because in the limit α→1, RE tends to Shannon’s entropy and the α-Gaussian distribution tends to the Gaussian distribution.

The leading order behavior near α=1 can be obtained directly from (43). The ensuing Taylor expansion gives

(46) $$\begin{array}{c} I_{\alpha }\left(Z_{\alpha }^{1,1}:Z_{\alpha }^{1,l}|Z_{\alpha }^{1,k}\right)\;=\;-\frac{l{\left(\alpha -1\right)}^{2}}{8}\;+\;O\left({\left(\alpha -1\right)}^{3}\right)\;, \end{array}$$

so, the point α=1 is a *stationary point* of $I_{\alpha }\left(Z_{\alpha }^{1,1}:Z_{\alpha }^{1,l}|Z_{\alpha }^{1,k}\right)$. This closes the proof.

## 4. Estimation of Rényi Entropy

### 4.1. RTE and Derived Concepts

From a data analysis point of view, it is not very practical to use the full joint processes $X_{n}^{\left(k\right)}$ and $Y_{n}^{\left(l\right)}$ (cf. the defining relation (10)) because (possibly) high values of *k* and *l* negatively influence the accuracy of estimation of RTE. In the following sections, we will thus switch to a more expedient definition of RTE given by

(47) $$\begin{array}{ccc} T_{\alpha ,Y\to X}^{R}\left(\left\{k\right\},\left\{m\right\},\left\{l\right\}\right)\; & = & \;H_{\alpha }\left(X_{{}_{n}}^{\{m\},+}|X_{n}^{\{k\},-}\right)\;-\;H_{\alpha }\left(X_{{}_{n}}^{\{m\},+}|X_{n}^{\{k\},-},Y_{n}^{\{l\},-}\right)\\ & = & \;I_{\alpha }\left(X_{{}_{n}}^{\{m\},+}:Y_{n}^{\{l\},-}|X_{n}^{\{k\},-}\right)\;, \end{array}$$

where $X_{n}^{\left\{k\right\},\Omega }$ is a subset of past (Ω=−) or future (Ω=+) values of $X_{t_{n}}$ with the number of elements equal to *k*, such that $\left\{k\right\}=\left\{\kappa_{1},\ldots ,\kappa_{k}\right\}$ is a set of indices and $X_{n}^{\left\{k\right\},\Omega }\equiv X_{t_{n\Omega \kappa_{1}}},X_{t_{n\Omega \kappa_{2}}},\ldots ,X_{t_{n\Omega \kappa_{k}}}$ is a selected subsequence of $X_{t_{n}}$, i.e., $n_{X}$-dimensional vectors. The same notational convention applies to $Y_{n}^{\left\{l\right\},\Omega }$ as a subsequence of $Y_{t_{n}}$, i.e., $n_{Y}$-dimensional vectors. In definition (47), we added a third parameter, *m*—the so-called *future step*. Though such a parametrization is often used in the literature on Shannon’s TE, cf., e.g., reference [17], we will (in the following) only employ m={1} so as to conform with the definition (10). In such a case, we will often omit the middle index in $T_{\alpha ,Y\to X}^{R}\left(\left\{k\right\},\left\{1\right\},\left\{l\right\}\right)$.

#### 4.1.1. Balance of Transfer Entropy

In order to compare RTE that flows in the direction from Y→X with the RTE that flows in the opposite direction X→Y, we define the *balance of transfer entropy*

(48) $$T_{\alpha ,Y\to X}^{R,\;\mathrm{balance}}\left(\left\{k\right\},\left\{l\right\}\right)\;=\;T_{\alpha ,Y\to X}^{R}\left(\left\{k\right\},\left\{l\right\}\right)\;-\;T_{\alpha ,X\to Y}^{R}\left(\left\{k\right\},\left\{l\right\}\right)\;.$$

#### 4.1.2. Effective Transfer Entropy

To mitigate the finite size effects, we employ the idea of a surrogate time series. To this end, we define the *effective transfer entropy*

(49) $$T_{\alpha ,Y\to X}^{R,\;\mathrm{effective}}\left(\left\{k\right\},\left\{l\right\}\right)\;=\;T_{\alpha ,Y\to X}^{R}\left(\left\{k\right\},\left\{l\right\}\right)\;-\;T_{\alpha ,Y^{\left(\mathrm{sur}\right)}\to X}^{R}\left(\left\{k\right\},\left\{l\right\}\right)\;,$$

where $Y^{\left(\mathrm{sur}\right)}$ stands for the randomized (reordered) time series—the surrogate data sequence. Such a series has the same mean, the same variance, the same autocorrelation function and, therefore, the same power spectrum as the original sequence, but (nonlinear) phase relations are destroyed. In effect, all the potential correlations between $X_{n}^{\left\{k\right\}}$ and $Y_{n}^{\left\{l\right\}}$ are removed, which means that $T_{\alpha ,Y^{\left(\mathrm{sur}\right)}\to X}^{R}\left(\left\{k\right\},\left\{l\right\}\right)$ should be zero. In practice, this is not the case, despite the fact that there are no obvious structures in the data. The non-zero value of $T_{\alpha ,Y^{\left(\mathrm{sur}\right)}\to X}^{R}\left(\left\{k\right\},\left\{l\right\}\right)$ must then be a byproduct of the finite data set. Definition (49) then ensures that spurious effects caused by finite *k* and *l* are removed. In our computations, we used the Fisher–Yates algorithm [66] together with Mersenne twister random generation algorithm [67] for the randomized surrogates. For a more technical exposition, see, e.g., refs. [68,69,70].

#### 4.1.3. Balance of Effective Transfer Entropy

Finally, we combined both previous definitions to form the *balance effective transfer entropy*

(50) $$\begin{array}{ccc} T_{\alpha ,Y\to X}^{R,\;\mathrm{balance},\;\mathrm{effective}}\left(\left\{k\right\},\left\{l\right\}\right)\; & = & \;T_{\alpha ,Y\to X}^{R,\;\mathrm{effective}}\left(\left\{k\right\},\left\{l\right\}\right)\;-\;T_{\alpha ,X\to Y}^{R,\;\mathrm{effective}}\left(\left\{k\right\},\left\{l\right\}\right)\\ & = & \;T_{\alpha ,Y\to X}^{R}\left(\left\{k\right\},\left\{l\right\}\right)\;-\;T_{\alpha ,Y^{\left(\mathrm{sur}\right)}\to X}^{R}\left(\left\{k\right\},\left\{l\right\}\right)\\ & - & \;T_{\alpha ,X\to Y}^{R}\left(\left\{k\right\},\left\{l\right\}\right)\;+\;T_{\alpha ,X^{\left(\mathrm{sur}\right)}\to Y}^{R}\left(\left\{k\right\},\left\{l\right\}\right)\;, \end{array}$$

to quantify the direction of flow of transfer entropy without finite size effects.

#### 4.1.4. Choice of Parameters *k* and *l*

The choice of the parameters *k* and *l* is essential to reliably analyze the information transfer between variables in a system. So, a natural question arises as to how one should choose such parameters.

The order of *k* and *l*, both in the RTE and Shannon’s TE, but also in approximating autoregression in the Granger case, is often (in practice) set rather arbitrarily at some moderately high number. In the literature, there are theoretical criteria for optimal choices of *k* and *l*—with no unique answer. In our numerical simulations, we employed two pragmatic criteria: (a) results should be stable under the increase of *k* and *l* and, additionally, (b) *k*, and *l* should be equal to—or higher than—those used in the literature for the analysis of Shannon’s TE in Rössler systems, e.g., references [18,22], so that we could make a comparison with the existence results. The chosen values ({k},{l})≡({k},{1},{l})=({0,1},{1},{0}) often well-satisfied both aforementioned conditions. In Section 6.3, it was sufficient to set {k}={0} and {l}={0}, in agreement with [18]. When a need has arisen to emphasize some finer details in the behavior of the RTE (cf. Figures 6 and 10), {k} was chosen to be {0,1,2,3,4} or even {0,1,2,3,4,5,6}.

## 5. Rössler System

### 5.1. Equations for Master System

In order to illustrate the use of RTE, we considered two unidirectionally coupled Rössler systems (oscillators). These often serve as testbeds for various measures of synchronization, including Shannon’s TE [71,72,73]. Rössler’s system is described by three non-linearly coupled partial differential equations

(51) $$\begin{array}{ccc} & & {\dot{x}}_{1}\;=\;-\omega_{1}\;\;x_{2}\;-\;x_{3}\;,\\ & & {\dot{x}}_{2}\;=\;\omega_{1}\;\;x_{1}\;+\;ax_{2}\;,\\ & & {\dot{x}}_{3}\;=\;b\;+\;x_{3}\left(x_{1}\;-\;c\right)\;, \end{array}$$

with four coefficients $\omega_{1}$, *a*, *b*, and *c*. Strictly speaking, only three coefficients are independent, as $\omega_{1}$ can be set to one by appropriately rescaling $x_{2}$. RS was invented in 1976 by O.E. Rössler [32] and it likely represents the most elementary geometric construction of chaos in the continuous systems. In fact, since the Poincaré–Bendixson theorem precludes the existence of (other than) steady, periodic, or quasi-periodic attractors in autonomous systems, defined in one- or two-dimensional manifolds, the minimal dimension for chaos is three [74]. The simplicity of the RS is bolstered by the fact that it only has one nonlinear (quadratic) coupling.

RS classifies as the *continuous (deterministic) chaotic system*, and more specifically as the *chaotic attractor*. The word “attractor” refers to the fact that whatever is the initial condition for the solution of the differential Equations (52), the trajectory x(t) ends up (after a short transient period) at the same geometrical structure (see Figure 5), which is neither a fixed point nor a limit cycle. This attractive geometrical structure is known as the Rössler attractor.

For future convenience, we will call the RS (51) as *driving* or *master system* and denote it as {X}.

### 5.2. Equations for the Slave System

In the following, we investigate RTE between two Rössler systems that are unidirectionally coupled in the variable $x_{1}$ via a small adjustable parameter ε. The corresponding second RS—*driven* or *slave system*, is defined as

(52) $$\begin{array}{ccc} & & {\dot{y}}_{1}\;=\;-\omega_{2}\;\;y_{2}\;-\;y_{3}\;+\;\varepsilon \left(x_{1}\;-\;y_{2}\right)\;,\\ & & {\dot{y}}_{2}\;=\;\omega_{2}\;\;y_{1}\;+\;ay_{2}\;,\\ & & {\dot{y}}_{3}\;=\;b\;+\;y_{3}\left(y_{1}\;-\;c\right)\;. \end{array}$$

Here, we fix the coefficients so that a=0.15, b=0.2, c=10.0, and frequencies $\omega_{1}=1.015$ and $\omega_{2}=0.985$, and initial conditions $(x_{1}\left(0\right),x_{2}\left(0\right),x_{3}\left(0\right))=\left(0,0,0\right)$ and $(y_{1}\left(0\right),y_{2}\left(0\right),y_{3}\left(0\right))=\left(0,0,1\right)$. This parametrization is adopted from reference [18] where Shannon’s TE between systems (51) and (52) was studied. In the following, we will denote the slave system also as {Y}.

### 5.3. Numerical Experiments with Coupled RSs

**Figure 4:**
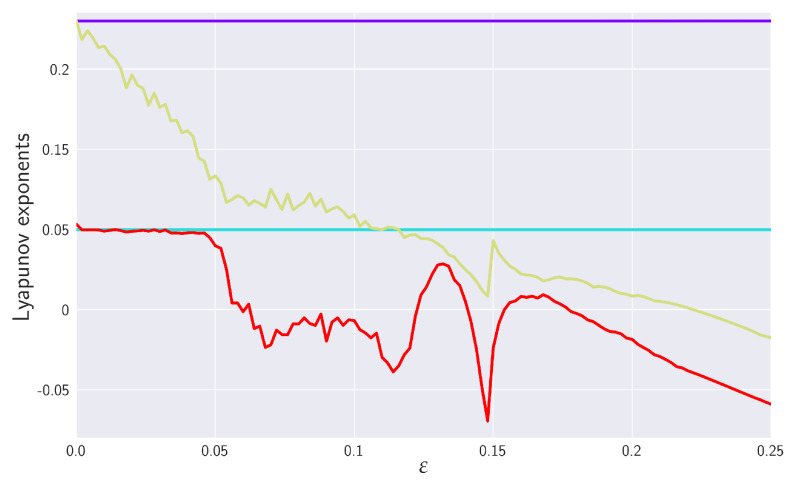
The two largest Lyapunov exponents of the master system (constant—violet and green) and the slave system (decreasing—red and yellow). So, for small ε, the signature of LE is ++00−−, while after synchronization, we end up with the signature +0−−−−. After synchronization, there is a “collaps” of the dimension, in the sense that the slave system is completely dependent on the master system, so that there is only one dimension (direction) in which there is an expansion. Accordingly, there is only one LE with a positive sign. The LEs are measured in nats per time unit.

**Figure 5:**
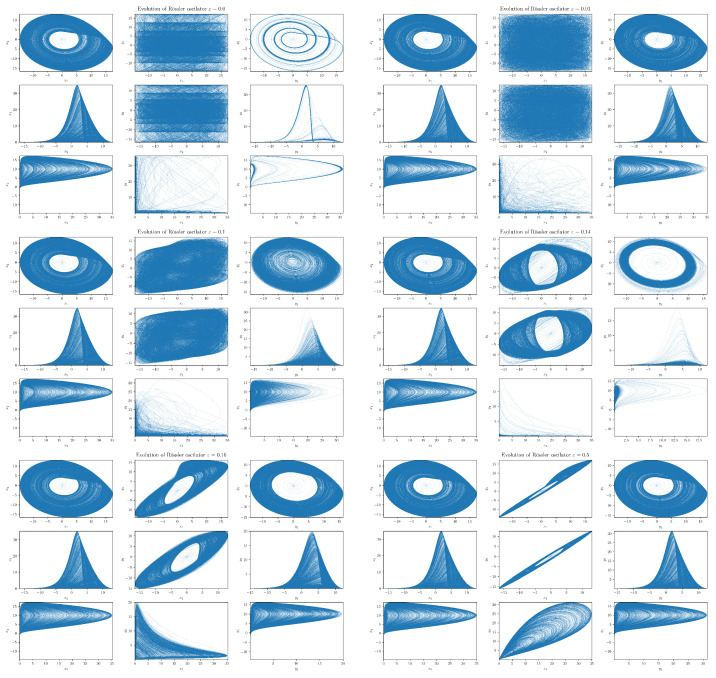
Projections of the RSs (51) and (52) on various planes. For each fixed ε, we depict nine figures that correspond (from top to bottom and left to right) to projections on the $x_{2}$-$x_{1}$, $x_{3}$-$x_{2}$, $x_{1}$-$x_{3}$, $x_{1}$-$y_{1}$, $x_{2}$-$y_{2}$, $x_{3}$-$y_{3}$, $y_{2}$-$y_{1}$, $y_{3}$-$y_{2}$, and $y_{1}$-$y_{3}$ planes. In the figure, we display, altogether, nine values of ε corresponding (from left to right and top to bottom) to ε=0,0.01,0.1,0.14,0.16 and 0.5. The initial values are chosen as $x_{1}\left(0\right),x_{2}\left(0\right),x_{3}\left(0\right)=0$, $y_{1}\left(0\right),y_{2}\left(0\right)=0$, and $y_{3}\left(0\right)=1$. Further projections for the transient region 0.12≲ε≲0.15 are shown in Figure 8. All RSs are depicted in the time window t= 10,000.

Before we embark on the RTE analysis, let us first take a look at the phenomenology of the coupled RSs (51) and (52) by means of simple numerical experiments. In our numerical treatment, we simulate coupled RSs by using the integration method, which is implemented in a package SciPy named solve_ivp with the LSODA option that exploits the Addams/BDF method, see, e.g., reference [75]. Projections of the ε-dependent RSs dynamics to various planes are presented in Figure 5. For visualization purposes, we used the toolkit Matplotlib [76] that exploits toolkit NumPy [77]. The sources are part of the Pyclits project [78]. In the future, the work can be rebased. The resulting data set analyzed consisted of 100,000 data points. To gain insight into the transient region, we chose shorter time lags in the data set generated from RS with 0.1≤ε≤0.15, namely, we reduced the time steps from 0.01 to 0.001. In parallel, we display in Figure 4 the behaviors of the corresponding Lyapunov exponents, as adapted from [22], which help to elucidate our discussion.

#### Projections

Instead of a conventional stereoscopic plotting, we found it more convenient (and illuminating) to focus on various plane projections of the coupled RSs. First, we noticed that, in Figure 5, the projections of RSs on the $x_{2}$-$x_{1}$, $x_{3}$-$x_{2}$, and $x_{1}$-$x_{3}$ planes do not depend on the coupling between systems (i.e., they are ε-independent), as expected, because the slave system (52) does not influence dynamics of the master system (51), which is autonomous (irrespective of ε). However, it is clear that signatures of the interaction between non-symmetrically coupled RSs (51) and (52) will show up in projections on the $x_{i}$-$y_{j}$ and $y_{i}$-$y_{j}$ planes.

Secondly, when the RSs are not coupled (i.e., when ε=0), we have two autonomous RSs—in fact, two strange attractors that differ only by values of their frequency coefficients and initial values. The autonomies of the respective RSs are clearly seen in projections on the $x_{i}$-$x_{j}$ and $y_{i}$-$y_{j}$ planes (cf. Figure 5). A different density of trajectories (in a given time window *t* = 100,000) can be ascribed to the frequency mismatch. Projections on the $x_{1}$-$y_{1}$ and $x_{2}$-$y_{2}$ planes show how the ensuing chaotic and (component-wise) uncorrelated trajectories fill their support regions. In particular, we can observe that on the background of densely packed chaotic trajectories, clear vertical stripes of dominantly-visited regions appear in the slave system. Vertical stripes are clearly visible because limit cycles in the autonomous slave system are far more localized than in the master system. The projection on the $x_{3}$-$y_{3}$ plane indicates that (most of the time) the master system orbits venture to the $x_{3}$ direction, the slave system orbits are in the vicinity of the $y_{1}$-$y_{2}$ plane, and vice versa.

By continuously increasing the coupling strength ε from the zero value, we can observe that, already, a small interaction significantly changes the evolution of the slave system. For instance, in Figure 5, we see that when ε=0.01, then the diffusive term $\varepsilon (x_{1}-y_{2})$ significantly disperses the limit cycles in the slave system. This is reflected not only in all projections on the $y_{i}$-$y_{j}$ planes but also in projections on the $x_{1}$-$y_{1}$ and $x_{2}$-$y_{2}$ planes. In the latter two cases, the diffusion causes that horizontal stripes to completely disappear. Finally, the projection on the $x_{3}$-$y_{3}$ plane does not change significantly from the ε=0 case.

When we further increase ε, we see that the behavior of the slave system starts to qualitatively depart from that of the master system. For ε, around 0.1, the slave system orbit diffuses to the region around the origin that is basically not visited (apart from an initial transient orbit) by the master system orbit (cf. projections on the $y_{i}$-$y_{j}$ planes). In addition, projections on the $x_{1}$-$y_{1}$ and $x_{2}$-$y_{2}$ planes disclose that the ensuing support areas are not filled anymore. In fact, we can see a development of a slant stripe structure. On the other hand, the projection on the $y_{3}$-$x_{3}$ plane reveals that the slave system orbits stop visiting regions further from $y_{3}=0$. A yet higher ε (around 0.14) orbit of the system {Y} first converges to a single limit cycle before it makes (again) a transition into a chaotic regime. Finally, we can observe that at ε∼0.14, the slave system rarely deviates far from $y_{3}=0$ and spends most of its time in the close vicinity of the $y_{1}$-$y_{2}$ plane—its evolution is “flattened”.

Moreover, at ε∼0.14, we can also notice that projections on the $y_{1}$-$x_{1}$ and $y_{2}$-$x_{2}$ planes underwent a change in topology (in fact, this happened already at around ε∼0.12). The onset of this “topological phase transition” is closely correlated with the behavior of the largest Lyapunov exponent (LE) of the slave system. In fact, coupled RSs altogether have six Lyapunov exponents. The ε=0 one has two autonomous RSs each with three LEs—one positive, one zero, and one negative (signature +0− is a typical hallmark of a strange attractor in three dimensions). While at ε=0, the signature of LEs is ++00−−, increasing ε all three LEs associated with {Y} decreasing (initially) monotonically, cf. Figure 4. After a transient negativity and a return to zero (red curve in Figure 4), the originally positive LE of the slave system monotonically decreases and the negative for ε≳0.15. In particular, we see that the critical value ε∼0.12 at which the “topological phase transition” occurs coincides with the value at which the largest LE of the system {Y} crosses zero.

What is particularly noteworthy is an abrupt (non-analytic) change in the behavior of LEs at the value ε∼0.145. At this value, the LE changes direction and starts to increase with increasing ε. The increase stops at ε∼0.15 when the yellow-colored LE in Figure 4 reaches (approximately) value zero, after which it monotonically decreases. Such a decrease also starts for the second red-colored LE, but at a slightly different value of ε.

For stronger interactions with 0.15≲ε≲0.2, we see (cf. Figure 5 with ε=0.16) that the slave system starts to approach the structure of the master system strange attractor (cf. $x_{i}$-$x_{j}$ and $y_{i}$-$y_{j}$ projections). From the tilt and thinning of projections on the $x_{1}$-$y_{1}$ and $x_{2}$-$y_{2}$ planes, one may deduce that the amplitude synchronizations in the $x_{1}$ and $y_{1}$ (as well as $x_{2}$ and $y_{2}$) directions increase. Projection on the $x_{3}$-$y_{3}$ plane shows that amplitudes in the $x_{3}$ and $y_{3}$ directions are also synchronized (being roughly a half-cycle behind each other).

Finally, for very strong interactions, e.g., for ε∼0.5, the synchronization is almost complete: the system {Y} basically fully emulates the master system’s behavior with both systems now being structurally identical (cf. $x_{i}$-$x_{j}$ and $y_{i}$-$y_{j}$ projections). Full synchronization is nicely seen in projections on the $x_{1}$-$y_{1}$ and $x_{2}$-$y_{2}$ planes. Note that the amplitudes in the $x_{3}$ and $y_{3}$ directions start to synchronize.

## 6. Numerical Analysis of RTE for Coupled RSs

In the previous section, we learned some essentials about the coupled RS (51) and (52). In order to demonstrate the inner workings of the RTE and to gain further insight into how the two RSs approach synchronization, we compute here the RTE for various salient situations, such as the RTE between the $x_{1}$- and $y_{1}$-component, between the $x_{1}$- and $y_{3}$-component, or RTE between the full master and slave system. In our numerical analysis, we employed the RE estimator introduced by Leonenko et al. [26]. Some fundamentals associated with this estimator are relegated to Appendix A.

### 6.1. Effective RTE between x_1_ and y_1_ Directions

**Figure 6:**
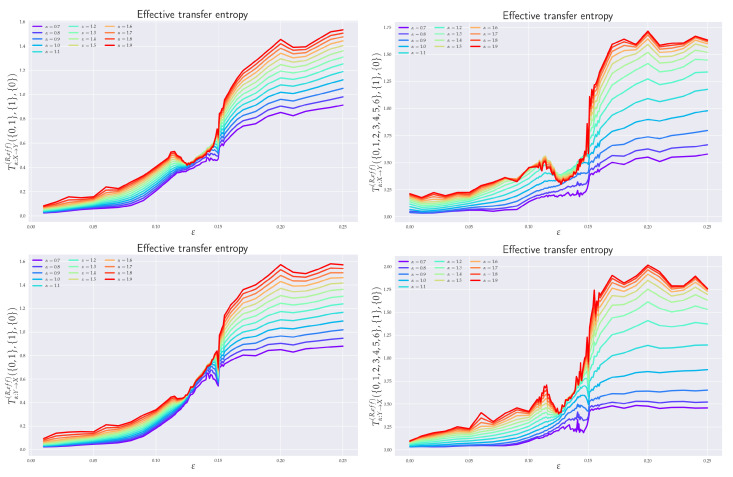
Effective RTE between $x_{1}$ and $y_{1}$ for two different histories of $x_{1}$, i.e., $T_{\alpha ,x_{1}\to y_{1}}^{R,\;\mathrm{effective}}\left(\left\{0,1\right\},\left\{1\right\},\left\{0\right\}\right)$, $T_{\alpha ,x_{1}\to y_{1}}^{R,\;\mathrm{effective}}\left(\left\{0,1,2,3,4,5,6\right\},\left\{1\right\},\left\{0\right\}\right)$, $T_{\alpha ,y_{1}\to x_{1}}^{R,\;\mathrm{effective}}\left(\left\{0,1\right\},\left\{1\right\},\left\{0\right\}\right)$, $T_{\alpha ,y_{1}\to x_{1}}^{R,\;\mathrm{effective}}\left(\left\{0,1,2,3,4,5,6\right\},\left\{1\right\},\left\{0\right\}\right)$, respectively, from left to right and top to bottom. RTE is measured in nats.

In order to understand the dynamics of the two coupled nonlinear dynamical systems (51) and (52) on their routes to synchronization, we first analyzed the effective RTE between the $x_{1}$ and $y_{1}$ components. Corresponding plots for different coupling strengths ε and different orders α are depicted in Figure 6. We can observe first that the effective RTE from $x_{1}$ to $y_{1}$ gradually increases with the increasing coupling strength until ε∼0.12. The regime between ε∼0.12 and ε∼0.15, as seen from Figure 5, corresponds to a transient synchronization behavior, which stabilizes only after ε∼0.15. This can also be seen from the behavior of the LEs at Figure 4. It should also be noted that the behavior of effective RTEs in the transient regime is apparently almost identical for all α in both $T_{\alpha ,x_{1}\to y_{1}}^{R,\;\mathrm{effective}}\left(\left\{0,1\right\},\left\{1\right\},\left\{0\right\}\right)$ and $T_{\alpha ,y_{1}\to x_{1}}^{R,\;\mathrm{effective}}\left(\left\{0,1\right\},\left\{1\right\},\left\{0\right\}\right)$. This would, in turn, indicate that the information transfer is the same across all sectors of the underlying probability distributions. Upon closer inspection though, such a highly correlated behavior will disappear when more historic data on {X} and {Y} are included (cf. $T_{\alpha ,x_{1}\to y_{1}}^{R,\;\mathrm{effective}}\left(\left\{0,1,2,3,4,5,6\right\},\left\{1\right\},\left\{0\right\}\right)$ and $T_{\alpha ,y_{1}\to x_{1}}^{R,\;\mathrm{effective}}\left(\left\{0,1,2,3,4,5,6\right\},\left\{1\right\},\left\{0\right\}\right)$ in Figure 6).

**Figure 7:**
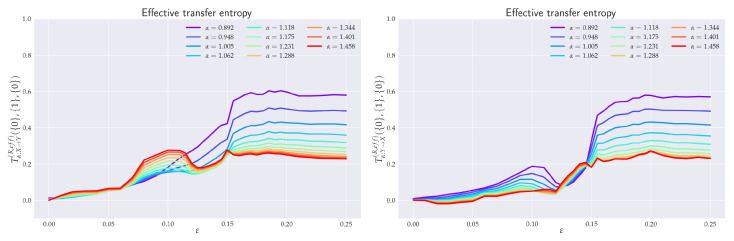
Effective transfer entropy for the full system ($n_{X}=3$ and $n_{Y}=3$) and for different values of α as functions of the coupling ε. We depict $T_{\alpha ,X\to Y}^{R,\;\mathrm{effective}}\left(\left\{0\right\},\left\{1\right\},\left\{0\right\}\right)$ (**left**) and $T_{\alpha ,Y\to X}^{R,\;\mathrm{effective}}\left(\left\{0\right\},\left\{1\right\},\left\{0\right\}\right)$ (**right**). RTE is measured in nats.

The same conclusion can be reached when the effective RTEs for the full six-dimensional systems are considered, cf. Figure 7.

**Figure 8:**
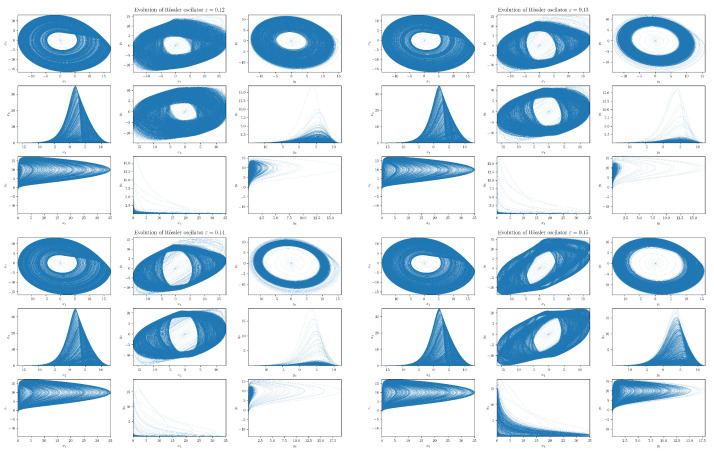
Four projections of the RSs (51) and (52) in the transient region 0.12≲ε≲0.15. Depicted are projections (from left to right, from top to bottom) with ε=0.12, 0.13, 0.14, and 0.15. With increasing ε, one can observe the contractions (measure concentrations) of the regions with denser orbit populations in the slave system. At the critical point ε=0.15, the contracted orbit regions abruptly expand and the slave system starts its way toward full synchronization with the master system (cf. also Figure 5). All RSs are depicted in the time window t= 10,000.

Nevertheless, from Figure 6, it can clearly be inferred that—in the transient region—strong correlations do exist, albeit not for all αs. In particular, one starts with the correlated flow for α≳1.2, which becomes stronger as ε increases. On the other hand, as ε approaches 0.15, the information flow decreases for α≲1. This can be seen clearly in both Figure 6 and Figure 7. At ε=0.15, the information flow abruptly increases for all αs. This is similar to a first order phase transition in statistical physics. In this respect, our “topological phase transition” would be more similar to a second order phase transition due to a smooth change in the entropic flow across the critical point ε=0.12. This scenario is also supported by Figure 8, where the actual behavior of the RS between the two critical points for four selected values of ε’s is depicted. Note, in particular, how the increase in the RTE for α≳1.2 (as well as the decrease of RTE for α≲1) are reflected in the contractions (measure concentrations) of the regions with denser orbit populations in the slave system. This, in turn, reinforces the picture that RTEs with higher αs describe the transfer of information between more central parts of underlying distributions, which, in this case, relate to higher occupation densities of the {Y} system orbit. From Figure 8, we can also note that, at the critical point ε=0.15, the contracted orbit regions abruptly expand and the slave system starts its way toward full synchronization with the master system. This is again compatible with the fact that the RTE abruptly increases for all αs at this point—i.e., all parts of underlying distributions participate in this transition and, consequently, the occupation density of the {Y} system orbit spreads. In this respect, point ε=0.15 represents the *threshold to full synchronization* while point ε=0.12 denotes the *threshold to transient behavior prior to full synchronization*. The latter can be identified with a phase synchronization threshold, which should be at (or very close to) this point [22].

**Figure 9:**
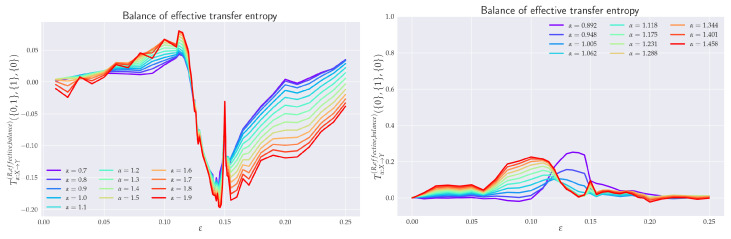
Balance of effective RTEs from $x_{1}$ to $y_{1}$ $T_{\alpha ,x_{1}\to y_{1}}^{R,\;\mathrm{balance},\;\mathrm{effective}}\left(\left\{0,1\right\},\left\{1\right\},\left\{0\right\}\right)$ (**left**, $n_{x_{1}}=1$ and $n_{y_{1}}=1$) and the balance of effective RTEs for the full system $T_{\alpha ,X\to Y}^{R,\;\mathrm{balance},\;\mathrm{effective}}\left(\left\{0\right\},\left\{1\right\},\left\{0\right\}\right)$ (**right**, $n_{X}=3$ and $n_{Y}=1$) with Y being $y_{1}$.

After the critical point ε∼0.15, both RSs enter full synchronization. In fact, the full synchronization starts when the information flow from all sectors of underlying distributions (i.e., for all αs) starts to be (almost) ε-independent and when $T_{\alpha ,X\to Y}^{R,\;\mathrm{balance},\;\mathrm{effective}}$ approach zero—so there is a one-to-one relation between the states of the systems, and the time series of the {X} system can be predicted from the time series {Y} system, and vice versa. Indeed, from Figure 6 (cf. also Figure 7 and Figure 9), we see that all $T_{\alpha ,Y\to X}^{R,\;\mathrm{effective}}$ proceed in a slow increase toward their asymptotic values in the fully-synchronized state.

### 6.2. Effective RTE between x_3_ and y_3_ Directions

**Figure 10:**
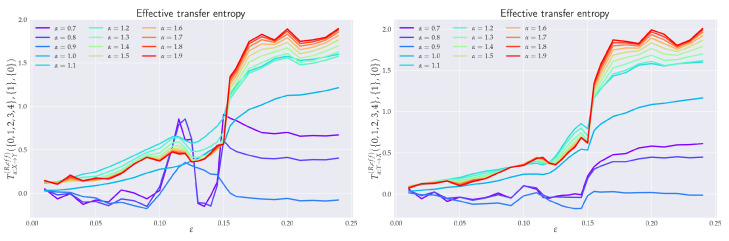
Effective RTE between $x_{3}$ and $y_{3}$ directions. From left to right: $T_{\alpha ,x_{3}\to y_{3}}^{R,\mathrm{effective}}\left(\left\{0,1,2,3,4\right\},\left\{1\right\},\left\{0\right\}\right)$ and $T_{\alpha ,y_{3}\to x_{3}}^{R,\mathrm{effective}}\left(\left\{0,1,2,3,4\right\},\left\{1\right\},\left\{0\right\}\right)$. Note a sudden increase in entropy transfer from the master to slave system at ε=0.12 (i.e., threshold to transient behavior) for α<1. RTE is measured in nats.

As already seen from Figure 5 and Figure 8, projections in the $x_{3}$-$y_{3}$ plane are particularly distinct. In Figure 10, we see the ensuing effective RTE between $x_{3}$ and $y_{3}$ directions.

What is particularly noticeable is a sudden increase in entropy transfer from the master to slave system at ε=0.12 (i.e., at the threshold to transient behavior) for α<1. No comparable increase is observed from slave to master. This, might be explained as an influx of information needed to organize the chaotically correlated regime that exists prior the (correlated) transient regime (cf. $x_{i}$–$y_{i}$ projections in Figure 5 and Figure 8). It should also be noticed that ordinary Shannonian TE (α=1) is completely blind to such an information transfer.

As for the transient region, we can observe that the effective RTE has qualitatively very similar behavior to the effective RTE between $x_{1}$ and $y_{1}$, namely a distinct decrease in the information transfer for α<1 and an increase for α>1. This again reveals a measure concentration. In this case, the orbit occupation density concentrates around the $y_{1}$-$y_{2}$ plane of the slave systems, cf. projections depicted in Figure 8. The situation abruptly changes at the synchronization threshold ε=0.15 after which the effective RTE approaches for each α a fixed asymptotic value that turns out to be the same for both $T_{\alpha ,x_{3}\to y_{3}}^{R,\mathrm{effective}}$ and $T_{\alpha ,y_{3}\to x_{3}}^{R,\mathrm{effective}}$.

### 6.3. Effective RTE for the Full System

In general, for a reliable inference, it is desirable that the conditioning variable in the definition or RTE (10) contains all relevant information about future values of the system or processes generating this variable in the uncoupled case. So, it should be a full three-dimensional vector *X* or *Y* in the case of RS. To this end, we display in Figure 7 the effective RTE for the full six-dimensional RS with information transfers in both X→Y and Y→X directions. Corresponding plots are depicted for different coupling strengths ε, different order αs, and different memories.

In particular, we can see that the information flow in the transient region starts after a brief decrease at around ε∼0.12 and sharply increases (in both directions) for α≳1.2. This implies that there is an increase in the correlating activity in between regions with higher occupation densities in both REs. The behavior depicted in Figure 8 can help us to better understand this situation. In particular, we see that in the transient region the {Y} system reshapes its orbit occupation density so that the ensuing measure concentrates more around its peak while its tail parts are thinner. In fact, Figure 8 also shows that this measure concentration increases until almost ε∼0.15. The measure concentration behavior is reflected by the decrease of the RTE for α≲1, i.e., decreasing information transfers between tail parts. This situation is even more pronounced when more memory is included in the effective RTEs, cf. both right pictures in Figure 7.

At the synchronization threshold ε=0.15, the information flow abruptly changes for all αs, with a particularly strong increase for α≲1. This indicates that the orbit occupation density of the {Y} system abruptly reshapes by lowering the measure concentrated around its peak and broadening it in tails, so that the tail parts may also enter the full synchronization regime.

Let us finally comment on the issue of bidirectional information flown for single-component RTEs. By envisioning the discretized versions of RSs, (51) and (52), one can see that RTE from the slave to the master system (e.g., between the $x_{3}$ and $y_{3}$ direction) cannot easily be zero. This is because $H_{\alpha }\left(X_{3,t_{n+1}}|X_{3,n}^{\left(k\right)},Y_{3,n}^{\left(l\right)}\right)$ in the relation (10) is not simply $H_{\alpha }\left(X_{3,t_{n+1}}|X_{3,n}^{\left(k\right)}\right)$. Note that due to the nonlinear nature of the coupled RSs, $y_{3}\left(t_{n}\right)$ depends both on $y_{1}\left(t_{n}\right)$ and $y_{1}\left(t_{n-1}\right)$ (via the third equation in (52)), while $y_{1}\left(t_{n}\right)$ depends on $x_{1}\left(t_{n}\right)$ and $x_{1}\left(t_{n-1}\right)$ (via the first equation in (52)); finally, $x_{1}\left(t_{n}\right)$ depends on $x_{3}\left(t_{n}\right)$ and $x_{3}\left(t_{n-1}\right)$ and also $x_{2}\left(t_{n}\right)$ and $x_{2}\left(t_{n-1}\right)$ (via the first equation in (51)); hence, $y_{3}\left(t_{n}\right)$ depends not only on $x_{3}\left(t_{n}\right)$, $x_{3}\left(t_{n-1}\right)$, $x_{3}\left(t_{n-2}\right)$ and $x_{3}\left(t_{n-3}\right)$ but also on historical values of $x_{2}$. In this way, $H_{\alpha }\left(X_{3,t_{n+1}}|X_{3,n}^{\left(k\right)},Y_{3,n}^{\left(l\right)}\left(X\right)\right)$ is not simply $H_{\alpha }\left(X_{3,t_{n+1}}|X_{3,n}^{\left(k\right)}\right)$, as other components beyond $X_{3,n}$ are also needed. Consequently, when single-component RTEs for RS are computed, we inevitably find a non-zero information transfer from the slave to the master system. The latter is not so much a problem of *k* and *l* but rather the fact that we did not account for all relevant components (we simply missed some information).

It is true that for a reliable inference, in general, it would be desirable to obtain a zero value in the uncoupled direction Y→X. This should be attained by proper conditioning—the conditioning variable should contain full information about future values of the system or processes generating this variable in the uncoupled case. So, it should be a three-dimensional vector X or Y for RS. Here, we computed effective RTE for the full six-dimensional system (vectors X and Y). From Figure 7, we can see that $T_{\alpha ,Y\to X}^{R,\;\mathrm{effective}}$ in the uncoupled direction stays at the zero value (particularly for larger values of α) up to close to the synchronization threshold (ε=0.12), while $T_{\alpha ,X\to Y}^{R,\;\mathrm{effective}}$ is distinctly positive there. So, RTE is a good *causal measure* only if the conditioning has a sufficient dimension (in our case, 3); otherwise, it can be viewed only as a measure of dependence.

### 6.4. Balance of Effective RTE

**Figure 11:**
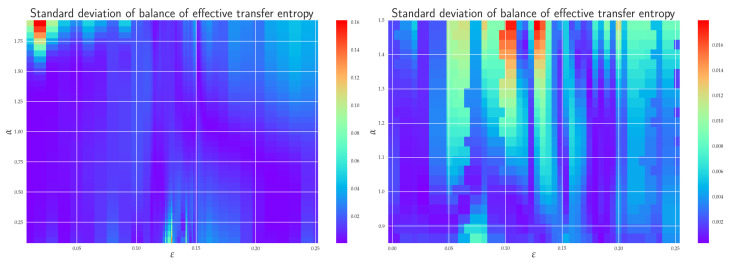
Dependence of standard deviation of the balance of effective RTEs $T_{\alpha ,x_{1}\to y_{1}}^{R,\;\mathrm{balance},\;\mathrm{effective}}\left(\left\{0,1\right\},\left\{1\right\},\left\{0\right\}\right)$ (**left**) and $T_{\alpha ,X\to Y}^{R,\;\mathrm{balance},\;\mathrm{effective}}\left(\left\{0\right\},\left\{1\right\},\left\{0\right\}\right)$ (**right**).

In order to quantify the difference between coupled (X→Y) and uncoupled (Y→X) information flow directions, we depict in Figure 9 the balance of effective RTEs between $T_{\alpha ,X\to Y}^{R,\;\mathrm{effective}}$ and $T_{\alpha ,Y\to X}^{R,\;\mathrm{effective}}$ for two different situations. Let us first concentrate on the balance of effective RTE $T_{\alpha ,x_{1}\to y_{1}}^{R,\;\mathrm{balance},\;\mathrm{effective}}\left(\left\{0,1\right\},\left\{1\right\},\left\{0\right\}\right)$. There, we can clearly see that before the synchronization threshold (“topological phase transition”), i.e., for ε≲0.12, we have $T_{\alpha ,x_{1}\to y_{1}}^{R,\;\mathrm{effective}}>T_{\alpha ,y_{1}\to x_{1}}^{R,\;\mathrm{effective}}$, which indicates the correct direction of coupling. The fact that for α>1.6 and ε≲0.04 one has $T_{\alpha ,x_{1}\to y_{1}}^{R,\;\mathrm{balance},\;\mathrm{effective}}\left(\left\{0,1\right\},\left\{1\right\},\left\{0\right\}\right)<0$ can be attributed to smaller reliability of the estimator in this region, cf. Figure 11 for estimation of ensuing the standard deviations. We can also observe that the synchronization threshold $T_{\alpha ,x_{1}\to y_{1}}^{R,\;\mathrm{balance},\;\mathrm{effective}}\left(\left\{0,1\right\},\left\{1\right\},\left\{0\right\}\right)$ changes sign and slowly return back to positive values in the fully synchronized regime. Similar behavior was reported in [22] for Shannon’s TE. Moreover, in this transient region, the effective RTEs have the same values irrespective of α, or, in other words, information transfer is the same across all sectors of the underlying probability distributions. This is akin to the behavior, which, in statistical physics, is typically associated with phase transitions—except for the fact that now we have a critical line rather than a critical point. However, as we already mentioned in the previous two paragraphs, this degeneracy is only spurious and will be removed by considering either the effective RTE for the full (six-dimensional) RS or longer memory.

After ε∼0.15, the approach to full synchronization proceeds at slightly different rates for different αs. This can equivalently be restated as saying that different parts of the underlying distributions enter synchronization differently. The dependence of the balance of effective RTE for the full (six-dimensional) system is shown on the right in Figure 9. Here, the behavior is less reliable for larger values of α (α≳1.2) and for smaller αs (α≲0.8), cf. Figure 11. In the region of reliable αs, the behavior is qualitatively similar to that of $T_{\alpha ,x_{1}\to y_{1}}^{R,\;\mathrm{balance},\;\mathrm{effective}}\left(\left\{0,1\right\},\left\{1\right\},\left\{0\right\}\right)$. On the other hand, apart from the region of a transient synchronization, we clearly have $T_{\alpha ,X\to Y}^{R,\;\mathrm{effective}}>T_{\alpha ,Y\to X}^{R,\;\mathrm{effective}}$, which implies the correct direction of coupling. The approach to full synchronization is also easily recognized—the RTEs saturate to constant values (i.e., information transfer is ε-independent) and both $T_{\alpha ,X\to Y}^{R,\;\mathrm{effective}}$ and $T_{\alpha ,Y\to X}^{R,\;\mathrm{effective}}$ start to approach each other. In this respect, RTEs with lower αs enter the synchronization regime slower than RTEs with larger αs. In other words, events described by the tail parts of the distributions $p(x_{n+1}|x_{n}^{\left(k\right)})$ and $p(x_{n+1}|x_{n}^{\left(k\right)},y_{n}^{\left(l\right)})$ (corresponding to α<1) will fully synchronize at higher values of ε than corresponding events described by central parts (α>1).

In passing, we might notice that since both $T_{\alpha ,X\to Y}^{R,\;\mathrm{effective}}$ and $T_{\alpha ,Y\to X}^{R,\;\mathrm{effective}}$ approach each other in the fully synchronized state, both the {X} and {Y} systems have to have the same underlying distributions (due to the reconstruction theorem for REs [21,34]) and, hence, they are indistinguishable, as one would expect.

## 7. Discussion and Conclusions

### 7.1. Theoretical Results

How one discerns ‘cause’ from ‘effect’ is the main question in many scientific areas. The seminal contribution of Wiener and Granger led to the so-called Granger causality principle and time series analysis method for inference of causality from experimental data. The traditional Granger causality method is based on linear autoregressive processes. However, nonlinear complex systems cannot be well-described by linear autoregressive models and require appropriate generalizations of the Granger causality method. One successful generalization stems from information theory, using a form of conditional mutual information, also known as transfer entropy. Shannon entropy-based TE has become a standard tool used for inferring causality from time series in all areas of science (including finance, climatology, neuroscience, etc.).

In this paper, instead of the Shannon entropy, we employed yet another information quantity, namely Rényi entropy. The ensuing RTE has the principal advantage that it is based on *a bona fide* information measure. In this way, one has a clear quantifier of the conveyed directional information (measured in bits or nats). Consequently, statements, such as: “the conveyed directional information from a tail part of the distribution is comparable with information from central part of distribution” or “information transfer is small/large (or good/bad)” are meaningful. RE is a measurable quantity; in principle, it can be measured directly (similar to Clausius entropy or Shannon entropy) without invoking the concept of the underlying distribution. This is because RE has an operational meaning given by various coding theorems. In practice, this is how RE is measured, e.g., in quantum optics (or more generally quantum information theory) [50,55]. In a conventional time series, one does not proceed this way because coding theorems (such as the Campbell coding theorem [44]) are difficult to implement for a large number of data.

As a proof of principle, we tested the concept of RTE on two unidirectionally coupled Rössler systems. The idea was to illustrate how the RTE can deal with such issues as synchronization and, more generally, causality in systems that are complex enough and yet amenable to a numerical analysis. Coupled RS is one of a handful of (simple) coupled chaotic systems that have been studied in the literature by means of Shannon’s TE. This point is particularly important because we needed a gauge to which we could compare our results (and to which our results should reduce for α=1). Despite the earlier applications of the RTE in bivariate (mostly financial) time series, many questions remained unanswered about how to properly qualify and quantify the results obtained. Here, we went ‘some way’ toward this goal.

First, we showed that the concept of the Granger causality is exactly equivalent to the RTE for Gaussian processes, which may, in turn, be used as a test of Gaussianity. This is because RTEs are in the Gaussian framework all the same, and, hence, the results should be α-independent. On the other hand, since the efficiency and robustness of RTE estimators crucially hinge on the parameter α employed, it might be (in many cases) easier to follow the information-theoretic route to Granger causality (provided the Gaussian framework is justified).

Second, we demonstrated that the equivalence between the Granger causality and RTE can also be established for certain heavy-tailed processes—for instance, for soft α-Gaussian processes. In particular, in this latter case, one could clearly see the connection between Granger causality, Rényi’s parameter α, and the heavy-tail power.

### 7.2. Numerical Analysis of RTE for Rössler Systems

In order to estimate the RTE, we employed the *ℓ*-nearest-neighbor entropy estimator of Leonenko et al. [26]. The latter is not only suitable for RE evaluation but it can also be easily numerically implemented to RTEs so that these can be computed almost in real time, which is relevant, e.g., in finance, regarding various risk-aversion decisions. Spurious effects caused by the finite size of the data set were taken into account by working with effective RTEs.

In order to gain further insight into the practical applicability and efficiency of the RTE, we tested it on two unidirectionally coupled Rössler systems—the master and slave system. To have a clear idea about what to expect, we first looked at the phenomenology of the coupled RSs by means of simple numerical simulations (presented in Figure 5). This was also accompanied by comparisons with Lyapunov exponents computed in references [18,22] and reproduced in Figure 4. In particular, we could clearly observe how the RSs synchronized with the increasing value of coupling strength. In this connection, we also identified critical values of coupling strengths at which *thresholds to transient behavior* (or the “topological phase transition”) and the *threshold to full synchronization* occurred.

More specifically, we were particularly interested in the transient region between chaotic correlation regimes and full synchronization, which had not as yet been discussed in the literature. To gain a better understanding of this region, we employed in the range ε∈[0.1,0.15] a higher frequency sampling, namely 0.001, in contrast to the standard 0.01 one used for other εs. The threshold to transient behavior was identified at the scale ε=0.12 where the positive LE crossed to negative values and where the projection on the $x_{1}$-$y_{1}$ and $x_{2}$-$y_{2}$ planes underwent topology changes (cf. Figure 5). From the point of view of RTEs, this threshold behavior was reflected in peaking the information flow in various directions. The increase in the effective RTE between $x_{1}$ and $y_{1}$ (in both directions) for α>1 was pronounced, in particular, which reflected the increase in orbit occupation density around the peak in the $y_{1}$-$y_{2}$ plane in the slave system. Even more marked was the high peak in information flow from $x_{3}$ to $y_{3}$ for α<1 (see Figure 10), which described an influx of information needed to “organize” chaotic correlations that existed between the $x_{3}$ and $y_{3}$ directions prior to ε≲0.12. Furthermore, the RTE was especially instrumental in understanding the measure concentration phenomenon in the transient regime. Finally, after a sharp “first-order-type” transition at the threshold of synchronization, the effective RTEs slowly approached their asymptotic values (distinct for each α) in the synchronized state. In addition, in the synchronized state, both $T_{\alpha ,X\to Y}^{R,\;\mathrm{effective}}$ and $T_{\alpha ,Y\to X}^{R,\;\mathrm{effective}}$ approached each other, which reveals that both {X} and {Y} systems have the same underlying distributions and, hence, they are indistinguishable.

As for the causality issue, we observed that the RTE is a good *causal measure* only if the conditioning has a sufficient dimension (in our case 3); otherwise, it is merely a *measure of dependence*. By employing effective RTE for the full system, we could reliably infer the coupling direction but only until ε≲0.12, i.e., until the threshold to transient behavior. After this value, the RSs started to synchronize, first partially (in the transient regime) and then fully ε=0.15. In fact, the full synchronization started when the information flows from all sectors of underlying distributions (i.e., for all αs) began to be (almost) ε independent and when $T_{\alpha ,X\to Y}^{R,\;\mathrm{balance},\;\mathrm{effective}}$ approached zero— so there was a one-to-one relation between the states of the systems and the time series of the {X} system could be predicted from the time series {Y} system, and vice versa; hence, one could not make any statement about the coupling direction.

We should also reemphasize that the standard deviation of the RTE importantly depends on α, cf. Equation (Figure 11). For instance, the balance effective RTE for the full system is around the transient region quite reliably described by 0.8≲α≲1.25, though the minimal noise value is not attained at α=1 (Shannon transfer entropy) but at α=1.16. Clearly, the α-dependence of fluctuations is generally dynamics-dependent, and in many interesting real-world processes, it is simply more reliable to utilize non-Shannonian TEs.

### 7.3. Conclusions

In this paper, we discussed the Rényi transfer entropy and its role in the inference of causal relations between two systems, i.e., in the identification of the driving and driven systems from the experimental time series. On the theoretical side, our focus was on understanding the connection between RTE and Granger causality. In particular, we proved that the Granger causality is entirely equivalent to the RTE for Gaussian processes. This generalizes the classic result of Barnett et al. [61] that is valid for Shannon’s TE. Furthermore, we have also shown how the Granger causality and the RTE are related in the case of heavy-tailed (namely α-Gaussian) processes. These results allow one to bridge the gap between autoregressive and Rényi entropy-based information-theoretic approaches.

On the experimental side, we illustrated the inner workings of the RTE by analyzing RTE between the synthetic time series generated from two unidirectionally coupled Rössler systems that are known to undergo synchronization. The route to synchronization was scrutinized by considering the effective RTE (and other derived concepts) between various master–slave components as well as between the full master and slave systems. We observed that with the effective RTE one could clearly identify a transient synchronization region (in the coupling strength), i.e., the regime between chaotic (master–slave) correlations and the synchronization threshold. In the transient region, the effective RTE allowed inferring the measure concentration for the orbit occupation density. It is noteworthy to mention that the latter cannot be deduced from Shannon’s TE alone.

We also saw that the direction of coupling and, hence, causality, could be reliably inferred only for coupling strengths ε<0.12 (the onset of the transient regime), i.e., when two RSs were coupled, but not yet fully. This is in agreement with earlier observations, cf., e.g., reference [22]. As soon as the RSs were synchronized, they produced identical time series; hence, there is no way to infer the correct causality relation solely from the measured data.

We conclude with a general observation—a clear conceptual advantage of information-theoretic measures in general, and RTE in particular, as compared to the standard Granger causality, are sensitive to nonlinear signal properties, as they do not rely on linear regression models. On the other hand, a clear limitation of RTEs, in comparison to the Granger causality, is that they are—by their very formulation—restricted to bivariate situations (though multivariate generalization is possible, it substantially increases dimensionality in the estimation problem, which might be hard to solve with a limited amount of available data). In addition, the RTEs often require substantially more data than regression methods.

## Figures and Tables

**Figure A1:**
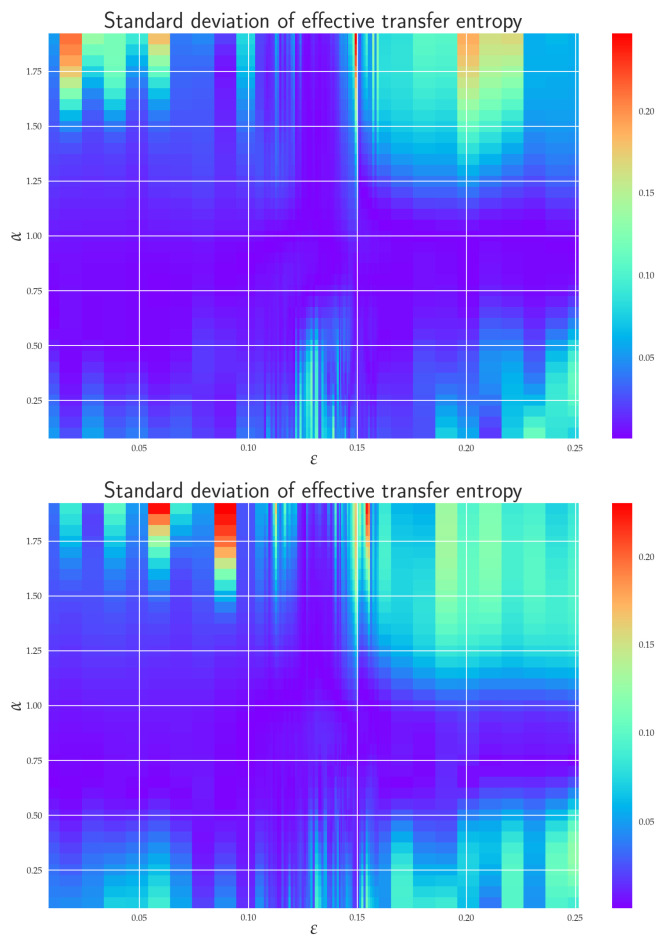
Standard deviation of the effective RTE between $x_{1}$ and $y_{1}$ $T_{\alpha ,x_{1}\to y_{1}}^{R,\;\mathrm{effective}}\left(\left\{0,1,2,3,4,5,6\right\},\left\{1\right\},\left\{0\right\}\right)$, and $T_{\alpha ,y_{1}\to x_{1}}^{R,\;\mathrm{effective}}\left(\left\{0,1,2,3,4,5,6\right\},\left\{1\right\},\left\{0\right\}\right)$.

**Figure A2:**
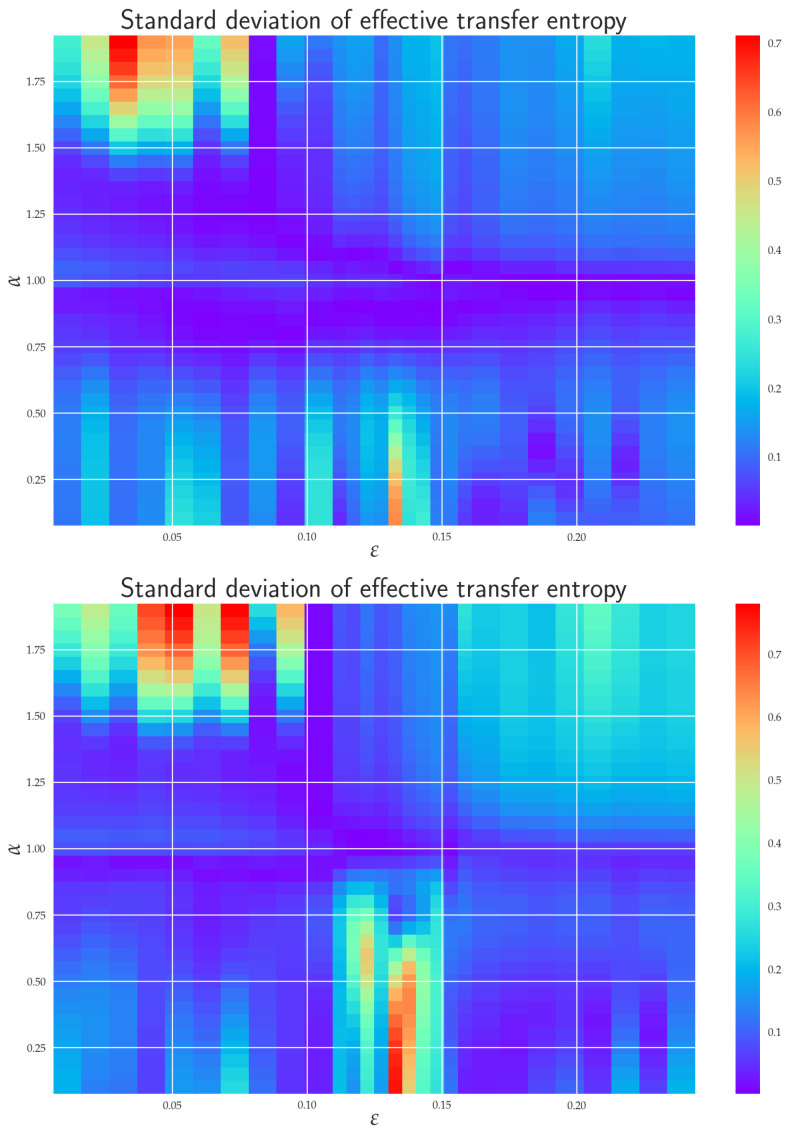
Standard deviation of the effective RTE between $x_{3}$ and $y_{3}$ for $T_{\alpha ,x_{3}\to y_{3}}^{R,\mathrm{effective}}\left(\left\{0,1,2,3,4\right\},\left\{1\right\},\left\{0\right\}\right)$, and $T_{\alpha ,y_{3}\to x_{3}}^{R,\mathrm{effective}}\left(\left\{0,1,2,3,4\right\},\left\{1\right\},\left\{0\right\}\right)$.

## Data Availability

Not applicable.

## Abbreviations

The following abbreviations are used in this manuscript:

RE	Rényi entropy
TE	transfer entropy
RTE	Rényi transfer entropy
PDF	probability density function
ITE	information-theoretic entropy
RS	Rössler system
KSE	Kolmogorov–Sinai entropy rate
LE	Lyapunov exponent

## Appendix A

Here, we provide a brief technical exposition of the RE estimator employed.

Finding good estimators for the RE is an open research area. The estimators for the Shannon entropy based on *ℓ*-nearest-neighbor in one-dimensional spaces were studied in statistics almost 60 years ago by Dobrushin [79] and Vašíček [80]. One disadvantage of these estimators is that they cannot easily be generalized to higher-dimensional spaces, so they are inapplicable to the TE calculations. Nowadays, there are many usable frameworks—most of them, of course, in the Shannonian setting (see reference [2] for a recent review). However, it is important to stress that the naive estimation of TE by partitioning of the state space is problematic [19] and that such estimators frequently fail to converge to the correct result [81]. In practice, more sophisticated techniques, such as kernel [82] or *ℓ*–nearest-neighbor estimators [83,84], need to be utilized. However, the latter techniques may bring about their own assumptions about the empirical distributions of the data (see [81] for a discussion about the issues involved).

In our work, we used the *ℓ*-nearest-neighbor entropy estimator for higher-dimensional spaces introduced by Leonenko et al. [26]. This estimator is suitable for RE and it can be effectively adapted and implemented by using formulas from the above-mentioned papers. In particular, the approach is based on an estimator of the RE from a finite sequence of *N* points that is defined as

(A1) $$\begin{array}{c} {\hat{H}}_{N,\ell ,\alpha }\;=\;\left\{\begin{array}{cc} \alpha \neq 1 & \;\;\;\;\log_{B}\left(\left(N-1\right)\cdot V_{m}\right)+\frac{1}{1-\alpha }\left[\log_{B}\frac{\Gamma \left(\ell \right)}{\Gamma \left(\ell +1-\alpha \right)}\right.\\ & \;\;\;\;\left.+\;\log_{B}\left(\frac{1}{N}\sum_{i=1}^{N}{\left(\rho_{\ell }^{\left(i\right)}\right)}^{m\left(1-\alpha \right)}\right)\right]\;\\ \alpha =1 & \;\;\;\;\log_{B}\left(\left(N-1\right)\cdot \exp\left(-\psi (\ell )\right)\cdot V_{m}\right)\\ & \;\;\;\;+\frac{m}{N}\sum_{i=1}^{N}\log_{B}\left(\rho_{\ell }^{\left(i\right)}\right) \end{array}\right.. \end{array}$$

Here, $\Gamma \left(x\right)$ is Euler’s gamma function, $\psi \left(x\right)=-\Gamma^{\prime }\left(x\right)/\Gamma \left(x\right)$ is the (negative) digamma function, $m=\dim X_{t}$ is the dimension of the data set space $X_{t}$, and $\rho_{\ell }^{\left(i\right)}$ is the distance from the data *i* to the *ℓ*-th nearest data counterpart using a metric in the space $X_{t}$. Moreover, $V_{m}$ is the size of the ball in space $X_{t}$ defined via the same metric. Finally, $\log_{B}$ is the logarithm with base *B* (we typically use B=e). In our computations, we employed the Euclidean metric, which has $V_{m}=\pi^{\frac{m}{2}}/\Gamma \left(\frac{m}{2}+1\right)$. Note that the estimator basically depends on *N*, i.e., the number of data in a data set and on *ℓ*, i.e., the rank of the nearest-neighbor used.

The advantages of the estimator (A1) in contrast to the standard histogram method are:

- It has relative accuracy for a small data set;
- It has applicability for high-dimensional data;
- The set estimators provide statistics for the estimation.

We should also note that, in contrast to other RE estimators, such as *fixed-ball* estimator [2], the estimator (A1) is not confined to any specific ranges of α values, though the efficiency of the estimator is, of course, α-dependent. We comment more on this point in Section 6. On the other hand, the disadvantage of this method involves the computational complexity of the algorithm and the complicated data container.

To calculate RTE and the related quantities (48)–(50), we apply the estimator Equation (A1). Ensuing estimators to (47)–(50)—let us call them generically X—become dependent on *ℓ* (i.e., the nearest-neighbor rank). We exploit this feature and define the mean value $\bar{X}$ and standard deviation $\sigma_{{}_{X}}$ with the Bessel correction, respectively, as

(A2) $$\begin{array}{ccc} & & \bar{X}\;=\;\frac{\sum_{\ell =n_{min}}^{n_{max}}X_{\ell }}{n_{max}-n_{min}+1}\;, \end{array}$$

(A3) $$\begin{array}{ccc} & & \sigma_{{}_{X}}\;=\;\sqrt{\frac{\sum_{\ell =1}^{n}{\left(X_{\ell }\;-\;\bar{X}\right)}^{2}}{n_{max}-n_{min}}}\;. \end{array}$$

Here, $n_{max}$ and $n_{min}$ are the highest and the lowest orders of the nearest data counterparts, respectively. Theoretically, we should use $n_{max}=M$, where *M* stands for the number of samples, but such a setup would require an enormous amount of computer memory to hold the distances.

In our calculations, we used $n_{max}=50$, which turned out to be a good compromise between accuracy and computer time. On the other hand, for $n_{min}$, we were a little bit restricted by the fact that $n_{min}$ influenced the interval of convergence of the estimator for various α (cf. discussion and proof in [26]). For instance, for ℓ=1, the estimator converged in the interval $\alpha \in [0,1+\frac{1}{2\dim\left(X_{t}\right)}]$, while for ℓ>1, one had $\alpha \in [0,\frac{\ell +1}{2}]$. For our particular purpose, it will suffice to set $n_{min}=5$, so that the interval of convergence will be α∈[0,3]. This will fully suit our needs.

## Appendix B

Here, we provide the heat maps for the relevant figures from the main text. These depict standard deviations (A2) and their dependencies on both α and ε.
